# Unveiling the power of Treg.Sig: a novel machine-learning derived signature for predicting ICI response in melanoma

**DOI:** 10.3389/fimmu.2025.1508638

**Published:** 2025-03-28

**Authors:** Yunlong Fan, Jiaman Yang, Xin Yang, Yulin Xie, Haiyang Li, Shuo Yang, Guanchao Sun, Ge Ge, Xiao Ding, Shengwei Lai, Yong Liao, Shuaifei Ji, Rongya Yang, Xingyue Zhang

**Affiliations:** ^1^ Department of Dermatology, The Seventh Medical Center of Chinese PLA General Hospital, Beijing, China; ^2^ Chinese PLA Medical School, Beijing, China; ^3^ Zhujiang Hospital, Southern Medical University or The Second School of Clinical Medicine, Southern Medical University, Guangzhou, China; ^4^ Department of Spine Surgery, The Third Hospital of Hebei Medical University, Shijiazhuang, China

**Keywords:** regulatory T cells, immunotherapy, scRNA-sequence, malignant melanoma, prognosis signature

## Abstract

**Background:**

Although immune checkpoint inhibitor (ICI) represents a significant breakthrough in cancer immunotherapy, only a few patients benefit from it. Given the critical role of Treg cells in ICI treatment resistance, we explored a Treg-associated signature in melanoma, which had never been elucidated yet.

**Methods:**

A new Treg signature, Treg.Sig, was created using a computational framework guided by machine learning, utilizing transcriptome data from both single-cell RNA-sequencing (scRNA-seq) and bulk RNA-sequencing (bulk-seq). Among the 10 Treg.Sig genes, hub gene STAT1’s function was further validated in ICI resistance in melanoma mice receiving anti-PD-1 treatment.

**Results:**

Treg.Sig, based on machine learning, was able to forecast survival outcomes for melanoma across training dataset and external test dataset, and more importantly, showed superior predictive power than 51 previously established signatures. Analysis of the immune profile revealed that groups with high Treg.Sig levels exhibited immune-suppressive conditions, with inverse correlations observed between Treg.Sig and anti-cancer immune responses. Notably, among the 10 Treg.Sig genes, hub gene STAT1 mutation harbored lower response rate in ICIs-treated cohort. Mechanistically, STAT1 impinged on ICI resistances by modulating the phenotypic switch in N2 neutrophil polarization in melanoma mice receiving anti-PD-1 therapy, which affects overall survival.

**Conclusion:**

The study developed a promising Treg.Sig signature that predicts ICI response of melanomas and could be used for selecting patients for immunotherapy. Meanwhile, our study potentially paves the way for overcoming immune resistance by targeting Treg-associated genes.

## Introduction

Surgery is the main treatment option for most melanoma and usually cures early-stage melanoma (1). Nevertheless, individuals with inoperable melanoma or those who experience distant metastasis (such as inoperable stage III, stage IV, or advanced melanoma) face limited choices for treatment. Anti-CTLA4 and anti-PD-1 antibodies, known as immune checkpoint inhibitors, work by activating CD8-positive T cells to kill cancer cells by targeting the malfunctioning immune system (2). The use of ICI therapy has drastically transformed the care of numerous types of cancer, especially advanced melanoma. In this type of cancer, around 50% of patients can experience tumor shrinkage and long-lasting responses to treatment for metastatic tumors, a significant improvement compared to the less than 10% success rate in the past (2). However, there are currently no precise biomarkers that are highly sensitive and specific in predicting the response to ICI treatment (3, 4). Limited clinical indicators, like neutrophil-to-lymphocyte ratio, serum lactate dehydrogenase, and BRAF mutation status, are utilized in choosing the first treatment for advanced melanoma patients, highlighting the necessity of novel biomarker researches to optimize patient selection (3, 5, 6).

Historically, biomarker studies have primarily concentrated on examining whole exome sequencing (WES) or RNA sequencing (RNA-Seq) from intact tumor tissue (bulk data), which only provides the overall genetic characteristics among a diverse cell population (7, 8). Consequently, predictive power of ICI biomarkers identified in these investigations was limited. Nevertheless, the advancement of scRNA-Seq technology allows the dissection of gene expression at individual cells resolution, thus enabling new biomarkers with improved predictive accuracy (9–11).

Regulatory T cells (Tregs), initially recognized by CD4+ CD25+ profile, dampen functions in anti-neoplastic immune cells, thus promoting tumor invasion (12). Previous studies have revealed that Tregs participate in cancer immune evasion and resistance (12–14). And studies have demonstrated a direct engagement of immunosuppressive CD4+ Tregs as a mechanism of immune evasion favored in melanoma (15–19). However, no direct clinical evidence has validated the negative association between Tregs and ICI outcomes.

This research involved integrated analysis using both single-cell RNA sequencing and bulk RNA sequencing to discover potential therapeutic markers for targeting Tregs. Utilizing machine learning, we developed a Treg.Sig tool to forecast the prognosis in skin cutaneous melanoma (SKCM) patients. Then, the clinical relevance of Treg.Sig was determined, and the PD-L1 expression, tumor immune dysfunction and exclusion (TIDE) score, and immune landscape underlying the Treg.Sig were further analyzed. Thereafter, a negative association between Treg.Sig and ICI outcomes in three ICI-treated cohorts was identified and validated. Finally, hub gene STAT1 was identified to play essential role in ICIs resistance. The discovery revealed that Treg.Sig has the potential to predict ICI outcomes with greater accuracy. In addition, our study provides new insights into the pathophysiology of SKCM, which could guide the development of customized treatments and potentially conquer immunotherapy resistance by targeting Treg.Sig genes.

## Methods

### Data source and acquisition

A total of 25 SKCM samples with scRNA-seq data, previously published by Li et al. (19), was downloaded for screening Tregs-associated marker genes. Data from The Cancer Genome Atlas (TCGA) bulk-seq and clinical data for 458 patients with SKCM were obtained to identify survival-related genes and create a signature. To confirm the predictive power of the developed signature, separate groups of patients were obtained from the GSE65904 datasets (n=209). Data from single-cell RNA sequencing of melanoma patients treated with carfilzomib or ICI were gathered from three separate datasets (GSE161801, GSE120575, and GSE189125) to assess the effectiveness of Treg.Sig in predicting the response to immunotherapy. The research made use of publicly accessible datasets that had received ethics approval from the original studies. The study was conducted according to the Helsinki declaration.

### Preprocessing of RNA-sequencing data

Analysis of single-cell RNA sequencing data was conducted with the R software tool “Seurat” (version 4.0.5) (20). Merged individual data and excluded low-quality cells based on gene detection criteria (nFeature_RNA >= 200 & nCount_RNA >= 500). Then, the “harmony” de-batching method was performed on 30 cases of SKCM scRNA-seq data. Following that, the scRNA-seq data that had been standardized underwent UMAP analysis for dimension reduction and were grouped using the “Seurat” tool in R software, with the parameters scale_factor=10000, nfeatures=3000, npcs=50, dims=30, and resolution = 0.2. Cell types were annotated based on markers using the SingleR package (version 1.0.0), and the expression patterns of each marker gene within clusters were analyzed with the “DotPlot” function in Seurat. Subsequently, potential marker genes for the Tregs cell cluster were pinpointed utilizing the FindAllMarkers feature.

To analyze bulk data, we used weighted co-expression network analysis (WGCNA) on the differentially expressed genes (DEGs) to find modules that are most relevant to Tregs. Initially, the correlations between genes and genes were computed in order to create a similarity matrix. Next, the optimal soft-thresholding power β was chosen using the pickSoftThreshold function within the “WGCNA” software package. By applying soft-thresholding, the similarity matrix was transformed to create a scale-free topology. Third, the adjacency matrix was transformed into a topological overlap matrix by similarity, and the corresponding dissimilarity was also calculated.

Finally, co-expression gene modules were determined by utilizing the R software tool “Dynamic Tree Cut” with a deepSplit value of 2, a minimum module size of 30, and a maximum block size of 20,000. Modules that were very alike were combined if the module eigengene height in the clustering was less than 0.25. As a result, the modules were connected to clinical characteristics in order to pinpoint the module with genes that were most pertinent to the Tregs.

### Construction and validation of prognostic Treg.Sig based on Tregs marker genes

**Figure 1:**
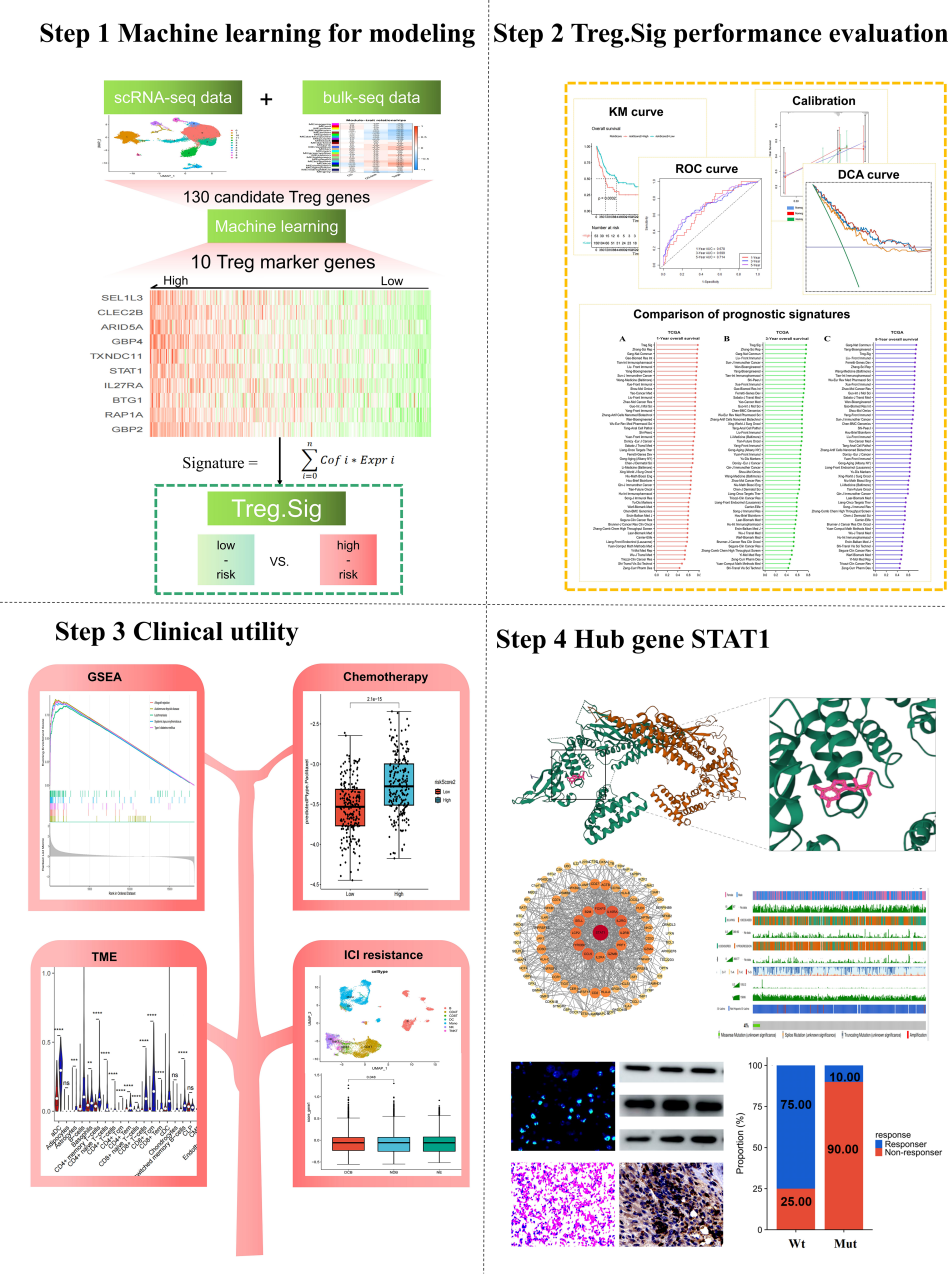
Workflow of the present study.

A novel machine learning-guided computational framework based on transcriptome data from scRNA-seq and bulk-seq was developed for identifying Treg.Sig as follows (**Figure 1**):

i. Candidates for Tregs marker genes were identified from the intersection cluster of bulk-seq and scRNA-seq.
ii. Prognostic Tregs marker genes were further screened out through Univariate Cox regression analysis conducted with the “survival” package in R software. Genes that had a P-value less than 0.05 were determined to be prognostic.
iii. Six different machine learning models, such as LASSO, Xgboost, SVM, Boruta, mRMR, and ReliefF, were employed to narrow down the variables. Each algorithm was chosen for its unique strengths and suitability for high-dimensional biological data. Hyperparameters (e.g., XGBoost: learning rate=0.1, max_depth=5, n_estimators=200; LASSO: α=0.032 via 10-fold cross-validation; SVM: C=1.0, γ=‘scale’) were optimized using grid search with 10-fold cross-validation. The final parameters minimized the cross-entropy loss for survival prediction. All models were trained and validated using nested 10-fold cross-validation to prevent data leakage. Outer loops partitioned data into training/testing sets, while inner loops optimized hyperparameters on the training subset only.
iv. A risk formula was created based on selected genes expression levels and weighting them with regression coefficients in multivariate Cox analysis, which was calculated as:

$$\mathrm{Treg.Sig}=\sum_{i=0}^{n}Cof\;i*TregsGene\;i$$

For performance evaluation:

i. Receiver operating characteristic (ROC) curve was created with the “survivalROC” tool in R (v1.0.3) to assess the predictive ability of Treg.Sig, measuring the area under the curve (AUC) at 1, 3, and 5 years.
ii. The survival predictive ability of Treg.Sig was estimated by generating a Kaplan Meier survival curve using the “survminer” package in the R software (version 0.4.9).
iii. The “survival” package in R software was used to conduct multivariate Cox regression analyses, which examined patient age, gender, stage, and Treg.Sig to determine if Treg.Sig could predict outcomes independently.
iv. The “timeROC” package in R software was utilized to create a ROC curve that varied with time, with AUC being employed to assess the predictive accuracy of Treg.Sig, age, and tumor stage.

To be clinically useful, a nomogram was created using the multivariate Cox regression coefficients for age, sex, cancer stage, and Treg.Sig. The nomogram’s predictive performance was validated by calculating the AUCs, examining the consistency between predicted 1-, 3-, and 5-year overall survival (OS) probabilities and actual observations with calibration curves, and measuring the clinical utility of the nomogram with decision curve analysis (DCA).

### Comparing Treg.Sig with other predictive gene signatures

To further estimate the prognostic performance of Treg.Sig, we compared Treg.Sig with other melanoma-specific signatures. 51 signatures were included (**Supplementary Table S2**), consisting of lncRNA, miRNA, and mRNA signatures, for comparison of the 1-year, 3-year, and 5-year AUC in external datasets GSE65904.

### Immune profile of Treg.Sig

64 types of immune infiltrating cells were calculated. The XCELL algorithm was used to quantify the infiltrating level of immune cells. Furthermore, the potential immunotherapy value of Treg.Sig was assessed using the immune proportion score (IPS), which measures the percentage of PD-L1 expression on immune cells, and the TIDE score, which indicates the expression signature of two main mechanisms of tumor immune evasion: T cell dysfunction and T cell exclusion. These two biomarkers could forecast the response to immune checkpoint inhibitors in individuals with cancer (21). The IPS of each individual with SKCM were acquired from The Cancer Immunome Atlas (TCIA) website (https://tcia.at/home), while the TIDE score was retrieved from the TIDE web platform (http://tide.dfci.harvard.edu).

Kyoko encyclopedia of genes and genomes (KEGG) and Gene ontology (GO) were used to analyze the immune enrichment of Treg.Sig marker gene with the help of R software packages: “ClusterProfiler” (version 4.0.5), “org.Hs.eg.db” (version 3.13.0), “ggplot2” (version 3.3.5), and “enrichplot” (version 1.12.3). The R software package “clusterProfiler” was utilized to conduct gene set enrichment analysis (GSEA) in order to explore the connection between Treg.Sig and immune response.

### Chemotherapeutic and immunotherapeutic response prediction of Treg.Sig

Currently, advanced SKCM patients are primarily treated with chemotherapy and ICI therapy. Our study selected four commonly used chemotherapeutic agents of SKCM, including AP_24534, Pazopanib, Paclitaxel, and Tivozanib. The half-maximal inhibitory concentration (IC50) chemotherapy drugs was estimated by utilizing the R software package “pRRophetic algorithm” and “pRRophetic” with data from the Cancer Genome Project (CGP) cell lines (22), in order to forecast the response to chemotherapy in groups categorized as high- and low-Treg.Sig.

Furthermore, the ICI response was predicted using SKCM patients from two GEO datasets (GSE120575 and GSE189125), with both scRNA-seq data and ICI treatment results. Meanwhile, the GSE161801 cohort comprised scRNA-seq data from pre-treatment melanomas receiving carfilzomib therapy. These data was also used to determine the potential value of Treg.Sig in predicting therapeutic responsiveness. The AddModuleScore function in the Seurat package was utilized to compute Treg.Sig scores in scRNA-seq data.

### Analysis of key regulatory gene and ICI response

Candidate differentially expressed genes (DEGs) were submitted to the STRING online database for analysis of protein-protein interactions (PPI), and the resulting network was visualized and hub genes were identified using Cytoscape software. Data from the HPA database (https://www.proteinatlas.org/) was used to analyze the protein expression of the hub gene STAT1 in SKCM tissues. To investigate the potential immunotherapy response of STAT1, six immune cell types were evaluated: activated dendritic cells (aDC), B cells, CD8 T cells, cytotoxic cells, macrophage cell, and T helper cells; along with three immune checkpoint molecules: PD-1, PD-L1, and PD-L2.

Moreover, the frequency of STAT1 mutations in melanoma was determined by analyzing the TCGA PanCancer Atlas studies (n=448) on cBioPortal (https://www.cbioportal.org/). In addition, in order to investigate the connection between STAT1 mutation and response to ICI treatment, data from clinical and tumor whole exome sequencing (WES) of melanoma patients from two cohorts treated with ICIs, including 38 samples from UCLA (Cell 2016) (23) and 64 samples from MSKCC (NEJM 2014) (24), were obtained for analysis. Every individual in the cohort treated with ICIs has received treatment with antibodies that target PD-1 and CTLA-4. In our research, melanoma patients with any nonsynonymous mutations in STAT1, such as missense, translation start site, nonstop, splice site, frameshift, and nonsense variants, were categorized under STAT1 mutations (STAT1 Mut). Conversely, patients without any STAT1 mutations were classified as having the STAT1 wild-type (STAT1 Wt).

### Cell culture and treatment

The Servicebio Technology Institute provided the B16-F10 melanoma cell line. These cells were cultured in RPMI-1640 supplemented with 10% FBS, 1% penicillin-streptomycin. All cells were grown in a 5% CO2 humid atmosphere at 37°C. For N1 neutrophils polarization, mouse primary neutrophils were treated with 1ug/ml LPS and 20 ng/ml IFN-gamma. For N2 neutrophils polarization, mouse primary neutrophils were isolated from femur and tibia, then purified by using MACS-based neutrophil isolation kit (Milteyni Biotec) following the manufacturer’s instructions, and then co-cultured with B16-F10 melanoma cells. For STAT1 inhibition, mouse primary neutrophils were cultured with 10uM Fludarabine (Flu, STAT1 inhibitor) or DMSO (vehicle).

### Tumor models and treatments

To implant subcutaneous tumors, B16-F10 melanoma cells were injected into the sides of 8- to 10-week-old mice using a saline solution containing 5×105 cells in 150µl. The size of the tumor was assessed weekly using a digital caliper and determined using the formula: volume = ½ (length × width × width) in millimeters cubed. Mice were sacrificed at end points of the study to analyze tumor weight, following the method outlined in a previous study (25). Mice received 200 µg of anti-PD-1 antibodies or IgG control per mouse through intraperitoneal injection weekly for specified weeks starting on day 7 post subcutaneous implantation of tumor cells. Some mice were injected intraperitoneally with Fludarabine (100 mg/kg per day) to inhibit the phosphorylation of STAT1. To perform adoptive transfer of N2 neutrophils, the N2 phenotype was acquired as outlined in the “Cell culture and treatment” section. The recipient mice were then given intra-tumor injections of N2 neutrophils (5 × 10^6^) that had been suspended in ice-cold PBS at a volume of 200 µl. Mice were sacrificed on the 28th day after implantation, and tumors were harvested for further experiments.

### Cell proliferation assays

To assess cell proliferation, the Cell Counting Kit-8 (CCK-8; Vazyme, Nanjing, China) method was utilized. B16-F10 melanoma cells were plated and then co-cultured with N2 neutrophils. Following this, each well received 10ul of CCK-8 solution and was incubated for 2 hours at 37°C, shielded from light. The viability of the cells was determined by measuring the absorbance at 450 nm with an enzyme-labeled meter (A33978, Thermo, USA), monitoring the changes at 24, 48, 72, and 96-hour intervals.

### Transwell assays

The transwell assay was conducted using a two-chamber invasion assay. In the upper chamber, 5×104 B16-F10 melanoma cells were seeded. Additionally, a bottom chamber with 5×104 N2 neutrophils was available. Following incubation, the cells beneath the filter were treated with 4% PFA, dyed with 0.1% crystal violet solution (Beyotime, Shanghai, China), and observed under a light microscope for counting.

### Reverse transcription-quantitative PCR

RNA extraction utilized TRIzol reagent (Invitrogen, Carlsbad, California), and the NanoDrop 2000 quantified the concentration of total RNA. cDNA synthesis followed, employing the ReverTra Ace qPCR RT Master Mix with gDNA remover (FSQ-301; Toyobo). The SYBR Green Kit (Vazyme, Nanjing, China) facilitated the quantitative PCR, using GAPDH as the internal standard. Primer designs were courtesy of Tsingke Biotech (Beijing, China), with sequences detailed in **Supplementary Table S3**.

### Western blotting and antibodies

Cell lysis was performed using RIPA buffer on ice, with protein concentrations determined via the BCA assay (Beyotime, Shanghai, China). Proteins from lysates underwent separation on 10% SDS-PAGE, followed by PVDF membrane transfer. Blocking occurred in 5% non-fat milk for an hour at ambient temperature before overnight primary antibody incubation at 4°C. Following triple washes with TBST, membranes were exposed to secondary antibodies for an hour at room temperature. Detection utilized an ECL kit.

### Immunofluorescence

Tissue sections that were fixed in formalin and embedded in paraffin were processed by removing wax, rehydrating, and retrieving antigens. In order to inhibit natural peroxidase activity, the sections were exposed to 3% H2O2 and then blocked with 3% BSA for a duration of 1 hour. Tissue samples were stained with anti-mouse antibodies targeting iNOS, Arg-1, Ly6G, and p-STAT1 overnight at 4°C, followed by secondary antibodies for one hour at ambient temperature, and DAPI to tag nuclei. An inverted fluorescence microscope captured the staining results, measured by ImageJ software.

### Flow cytometry

Cell suspensions from cancer samples (n=3) were washed with PBS and permeabilized using the eBioscience™ Foxp3/Transcription Factor Staining Buffer Set for flow cytometry analysis. Proceed with the steps outlined in the manufacturer’s guidelines (catalog. 00-5523-00). Antibodies that had been conjugated were introduced and left to incubate for one hour at ambient temperature. The antibodies used included Alexa Fluor™ 700-CD11b (eBioscience, 56-0112-80, 1:100), FITC-Ly-6g (eBioscience, 11-5931-82, 1:400), PE-iNOS (eBioscience, 12-5920-80, 1:300), and APC-Arginase (eBioscience, 17-3697-80, 1:100). A flow cytometry recorded cell fluorescence, with FlowJo software analysis and appropriate control antibodies ensuring data accuracy.

### Statistical analysis

Pearson correlation analysis assessed continuous variables, while group differences were evaluated using two-tailed t-tests or one-way ANOVA. Cox hazards regression model identified independent prognostic factors, with significance set at p<0.05. Data analysis and visualization were conducted with R software version 4.1.0 (http://www.R-project.org).

## Results

### Identifying Tregs marker genes by scRNA-seq and bulk-seq

Following the preprocessing of scRNA-seq data using strict quality control measures as indicated (**Supplementary Figure S1**), 64,071 infiltrating immune cells of high quality were extracted from 30 individuals diagnosed with melanoma, encompassing patients at different stages of the disease and with varied treatment backgrounds. Subsequently, UMAP was performed using Seurat to reduce the dimensionality and identified 12 cell clusters (**Supplementary Figure S2A**), which was subsequently identified as specific cell types through annotation using the SingleR package in R. T-cells, myeloid cells, B-cells, fibroblasts, and NK cells were identified as the main cell types in the study (**Supplementary Figure S2B**). Furthermore, the accuracy of the automated Treg cell cluster annotation was further confirmed by manually validating the Tregs cluster using Treg cell markers (FOXP3, IL2RA, TIGIT, IKZF2, BATF, TNFRSF4, and TNFRSF9) (26) (**Supplementary Figures S2C–F**). Then, 1041 genes related to Tregs were obtained using the Findmarkers function in Seurat, and were considered as candidate genes for further analysis.

WGCNA is a useful bioinformatics technique for analyzing gene correlation patterns in microarray or RNA sequencing data. It detects modules containing genes that are closely related, establishes connections between modules and external sample characteristics, and pinpoints potential biomarkers or targets for treatment. The soft-thresholding power value was a crucial parameter that impacted the autonomy and average connectivity level of co-expression modules. The optimal soft-thresholding exponent was determined based on a scale-free topology fit index of 9 (**Supplementary Figure S3A**). A power value of 6 was selected, which clustered all genes into 24 modules. The hierarchical cluster tree displayed by **Supplementary Figure S3B** illustrated 24 modules of co-expressed genes analyzed using WGCNA, where genes with similar expression profiles were clustered together. Genes within the identical color block exhibit comparable expression patterns and could potentially share functional relationships. Analyzing the clinical data revealed a strong correlation between the blue module and Tregs, with a Pearson correlation coefficient of 0.47 and a p-value of 6e-27 (**Supplementary Figure S3C**). Furthermore, a strong relationship was observed between gene co-expression in the blue module and two clinical features: OS state (Pearson Correlation = -0.18, P=2e-04) and OS times (Pearson Correlation = 0.13, P=0.007) (**Supplementary Figure S3C**). This indicates that, among the 24 modules, genes in the blue module, consisting of 398 genes, are highly associated with Tregs and tumor recurrence. Further analysis was conducted on the relationship between 398 module membership and gene significance for Tregs, showing a Pearson correlation coefficient of 0.4 (P=1e-16) (**Supplementary Figure S3D**). In conclusion, 398 candidate genes of Tregs were obtained through the bulk sequence.

Finally, 130 intersected candidate genes were identified from scRNA-seq and bulk-seq (**Supplementary Figure S4A**), and were further used to screen out the most valuable Treg marker genes.

### Development of Treg.Sig

Univariate Cox regression identified the prognostic value of 130 potential genes for feature selection. A total of 114 Tregs genes were found to have a significant correlation with OS (P<0.05) in **Supplementary Table S1**. Next, the most prognostic genes were further screened out using six feature selection algorithms, all of which were widely used in the machine learning literature. Ten hub genes, including “GBP2”, “RAP1A”, “BTG1”, “IL27RA”, “STAT1”, “TXNDC11”, “GBP4”, “ARID5A”, “CLEC2B”, and “SEL1L3”, were identified for the construction of the prognostic model (**Supplementary Figure S4B**).

Before modeling, we measured the reliability of above 10 hub genes by GO and KEGG enrichment analyses. As illustrated in **Supplementary Figure S4C**, these Tregs marker genes were associated with immune characteristics, including positive regulation of T-helper 1 type immune response, regulation of T-helper 17 type immune response, and IL-17 production. **Supplementary Figure S4D** displays the top 10 KEGG pathways that are enriched, primarily related to immune functions, including the NOD-like receptor signaling pathway, Th17 cell differentiation, JAK-STAT signaling pathway, chemokine signaling pathway, Kaposi sarcoma-associated herpesvirus infection, and inflammatory bowel disease. Furthermore, unsupervised machine learning (consensus clustering) indicated that for cluster k values of 2 or 3, analyses of OS rates showed different survival outcomes among the subtypes (**Supplementary Figures S4E, F**). These enrichment terms and unsupervised machine learning confirmed that a combination of machine learning and scRNA- and bulk- sequence data was reliable for Tregs marker screening.

Conducting a multivariate Cox analysis yielded the coefficients associated with the development of Treg.Sig. The detailed formula was as follows: Treg.Sig = (-0.0119 ∗expression value of GBP2) + (-0.0839 ∗expression value of RAP1A) + (-0.0690 ∗expression value of BTG1) + (-0.0829 ∗expression value of IL27RA) + (-0.0294 ∗expression value of STAT1) + (-0.1577 ∗expression value of TXNDC11) + (-0.1128 ∗expression value of GBP4) + (-0.2128 ∗expression value of ARID5A) + (-0.1169 ∗expression value of CLEC2B) + (-0.0492 ∗expression value of SEL1L3).

Then, the Treg.Sig score for each patient was calculated using the above formula. Patients were categorized into high-risk (n=229) and low-risk groups (n=229) based on the median cutoff point of 0.03. **Supplementary Figure S5A** displays the distribution of risk scores and survival status for each patient. The heatmap exhibited detailed expression level of the enrolled ten genes (**Supplementary Figure S5B**). In the TCGA-SKCM dataset, Kaplan-Meier survival analysis indicated that patients at high risk had notably poorer OS (P<0.001) (**Supplementary Figure S5C**). The prognostic accuracy of Treg.Sig was confirmed by time-sensitive ROC curves showing AUC values of 0.777, 0.762, and 0.721 for 1, 3, and 5 years of OS, respectively (by DeLong’s test; **Supplementary Figure S5C**). The performance of Treg.Sig was externally assessed in GSE65904 cohort to validate its robustness. Kaplan-Meier analysis showed that patients with a low Treg.Sig score had notably improved OS (P=0.0032) (**Supplementary Figure S5D**). The validation cohort also demonstrated strong performance in the ROC curves of the risk score (**Supplementary Figure S5D**). The Treg.Sig was identified as a significant factor in the multivariate Cox regression model, with a hazard ratio of 0.421 (95%CI 0.315–0.526, P<0.001), independent of age, gender, and clinical stage (**Supplementary Figure S5E**). Furthermore, Treg.Sig worked better than these factors in comparative ROC curves (**Supplementary Figure S5F**).

To assist clinicians in predicting the OS of SKCM patients, a nomogram was developed that integrated Treg.Sig with age, gender, and tumor stage for practical use. The new nomogram evaluated the chances of survival at 1, 3, and 5 years (**Supplementary Figure S6A**). The ROC curves, calibration, and DCA plots were drawn to evaluate the reliability of the nomogram. In the internal validation cohort, the AUCs in the ROC curve for predicting OS at 1, 3, and 5 years were 0.790, 0.809, and 0.815, as shown in **Supplementary Figure S6B**. In the GSE65904 external validation cohort, the values were 0.761, 0.756, and 0.738, respectively (by DeLong’s test; **Supplementary Figure S6C**). Furthermore, the calibration plots showed a line segment near the 45 degree, suggesting a strong agreement between the prediction and observation in the internal validation cohort (**Supplementary Figure S6D**) and GSE65904 external validation cohort (**Supplementary Figure S6E**). And the DCA showed that Treg.Sig would offer a robust net benefit in internal validation cohort (**Supplementary Figure S6F**) and GSE65904 external validation cohort (**Supplementary Figure S6G**).

### Comparison of prognostic value between Treg.Sig and published signatures

**Figure 2:**
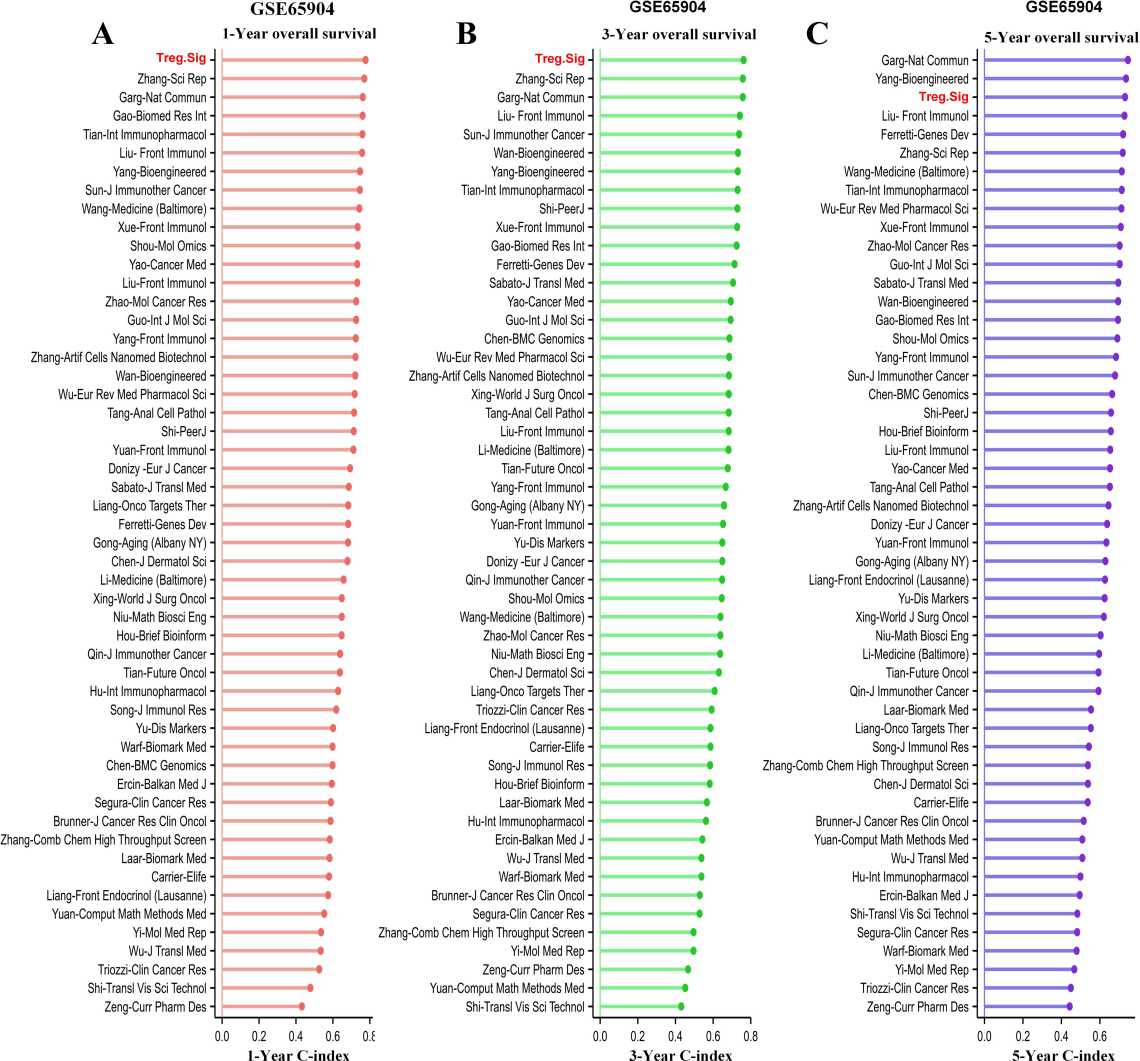
Comparison between Tregs signature and previous models. The 1-year C-index **(A)**, 3-year C-index **(B)**, and 5-year C-index **(C)** of Tregs signature and other models in the GSE65904 cohort.

Over the last couple of years, there has been an increase in the development of various gene-expression signatures that have different predictive abilities. The predictive power of Treg.Sig was examined and compared to 51 signatures (**Supplementary Table S2**), encompassing both lncRNA- and mRNA-derived signatures, to assess AUC at 1-, 3-, and 5-year OS. The 51 signatures showed connections to various biological characteristics, such as immune cell infiltration, cell death processes, cellular transformation, hypoxia, genetic modifications, N6-methyladenosine, etc. The Treg.Sig exhibited superior performance compared to all other signatures in predicting 1-year and 3-year OS in the GSE65904 dataset (**Figure 2**).

### Validation of the Treg.Sig in different clinical subgroups

The Treg.Sig was further validated in several SKCM subgroups stratified by different clinical characteristics, included genders, OS, and tumor stages. In particular, the elevated risk score correlated with higher clinical stage and OS status in SKCM (**Supplementary Figures S7A–C**). Furthermore, the OS was notably poorer in the high-risk cohorts compared to the low-risk cohorts, either in the early clinical stage (P=2.3×10−4) (**Supplementary Figure S7D**) or advanced clinical stage (P<0.0001) (**Supplementary Figure S7E**) SKCM subgroups. Clark’s level of invasion significantly predicts tumor invasion in melanoma (27). Likewise, we found that Treg.Sig exhibited a robustly predictive power, whether in Clark 1, 2, and 3 subgroups (P=0.0046) (**Supplementary Figure S7F**), or Clark 4 and 5 subgroups (P=0.0008) (**Supplementary Figure S7G**).

### Treg.Sig is associated with immune profiles

The T lymphocytes were crucial in the immune response against the tumor. Consequently, we examined how Treg.Sig is associated with immune cell infiltration and immunotherapy response in patients with SKCM. Initially, we examined how the signature is connected to 64 different types of immune cells that infiltrate. xCELL algorithms demonstrated that SKCM patients with high-risk scores had a higher proportion infiltration of mesenchymal stem cells, osteoblast, and NKT cells. Despite this, their composition included fewer aDC cells, B cells, CD4+ memory T cells, CD4+ naive T cells, CD8+ Tcm, CD8+ Tem, cDC cells, iDC cells, M2 macrophages, memory B−cells, monocytes, pDC cell, Th2 cells, and Tregs, suggesting an inflamed yet somewhat immunosuppressive environment (**Supplementary Figure S8A**).

In melanoma cancer, T cells that have infiltrated the tumor exhibit anti-tumor immune responses. Therefore, utilizing T cell-focused immunotherapy approaches could offer a novel opportunity to enhance cancer treatment (28). Thus, we conducted additional analysis to explore the connection between Treg.Sig and immunotherapy by examining the association between Treg.Sig and established immunotherapy markers. Initially, we utilized the TIDE online algorithm to forecast the likelihood of response to ICI therapy. While low-Treg.Sig patients exhibited higher TIDE scores (Chi-square test, P=0.0038; **Supplementary Figure S8B**), this apparent paradox may reflect compensatory immune evasion mechanisms in tumors with baseline low immunosuppression. Considering the TIDE score’s predictive performance is context-dependent, we included the immune proportion score (IPS) in our analysis to provide a more comprehensive assessment, as it is a more precise biomarker for the protein expression of PD-L1. Predictably, patients with low risk exhibited notably elevated levels of PD-L1 protein expression compared to high-risk patients in the ctla4_neg_pd1_neg group (P=0.011) (**Supplementary Figure S8C**), ctla4_neg_pd1_pos group (P<0.0001) (**Supplementary Figure S8D**), ctla4_pos_pd1_neg group (P<0.0001) (**Supplementary Figure S8E**), and ctla4_pos_pd1_pos group (P<0.0001) (**Supplementary Figure S8F**). Overall, these findings suggested that Treg.Sig correlated with the infiltration of immune cells and the immune status of tumors in the tumor microenvironment.

### Biological pathways related to the Treg.Sig

Due to the immune-related characteristics of the Treg.Sig, we then tended to dig into its underlying mechanism of action. Initially, we conducted a correlation analysis to identify the genes that are highly associated with the Treg.Sig. As illustrated in the **Supplementary Figures S9A** and **B**, a total of 438 genes were found to have a positive relationship, while 28 genes had a negative relationship, including immune checkpoints such as CTLA-4, LAG-3, TIGIT, CD8A, CD163, and more. Subsequently, GSEA analysis was conducted on these genes using the R software package “clusterProfiler”, indicating their involvement in immune-related processes like allograft rejection, autoimmune thyroid disease, leishmaniasis, systemic lupus erythematosus, and type I diabetes mellitus (**Supplementary Figure S9C**). Taken together, our data indicated that Treg.Sig participates in the immune related response. Thus, studies to detect further potential value of Treg.Sig are necessary.

### Treg.Sig was predictive of chemotherapy response and ICI resistance

Subsequently, we examined the feasibility of utilizing Treg.Sig to inform systemic treatments. Initially, the pRRophetic algorithm was utilized to estimate IC50 values in order to forecast the varying responses to chemotherapy in Treg-associated high- and low-risk groups. According to the Cancer Genome Project (CGP) database, a decreased Treg.Sig risk score was associated with increased sensitivity to four different chemotherapy drugs (AP_24534, Pazopanib, Paclitaxel, Tivozanib) based on the Wilcoxon test results (all P< 0.0001) (**Supplementary Figures S10A–D**). Therefore, low Treg.Sig score was correlated with high chemotherapy sensitivity.

We then confirmed the effectiveness in various immunotherapy datasets, such as GSE161801 (carfilzomib), GSE120575 (anti PD1 or/and CTLA4), and GSE189125 (anti PD-1). Melanoma patients exhibited lower Treg.Sig score after new proteasome inhibitors therapy–carfilzomib (Wilcoxon test, P=1.2x10-7) in the GSE161801 scRNA-Seq cohort (**Supplementary Figures S10E, F**). Similarly, individuals who responded to anti PD1 or/and CTLA4 treatment (GSE120575) exhibited significantly lower Treg.Sig scores than those who did not respond (Wilcoxon test, P<0.0001), suggesting a negative correlation between Treg.Sig score and ICI effectiveness (**Supplementary Figures S10G, H**). In the GSE189125 anti PD-1 cohort, the durable clinical benefit (DCB) group showed significantly lower risk scores than the no durable benefit (NDB) group (Wilcoxon test, P=0.048), suggesting that patients with lower risk were more responsive to immunotherapy than those with higher risk (**Supplementary Figures S10I, J**). The findings indicated that individuals with low-Treg.Sig scores have a higher chance of responding well to immunotherapy, suggesting that Treg.Sig could serve as a valuable biomarker for identifying SKCM patients who could benefit from immunotherapy.

### Key Treg.Sig gene STAT1 is associated with patient survival outcome and immune checkpoint

**Figure 3:**
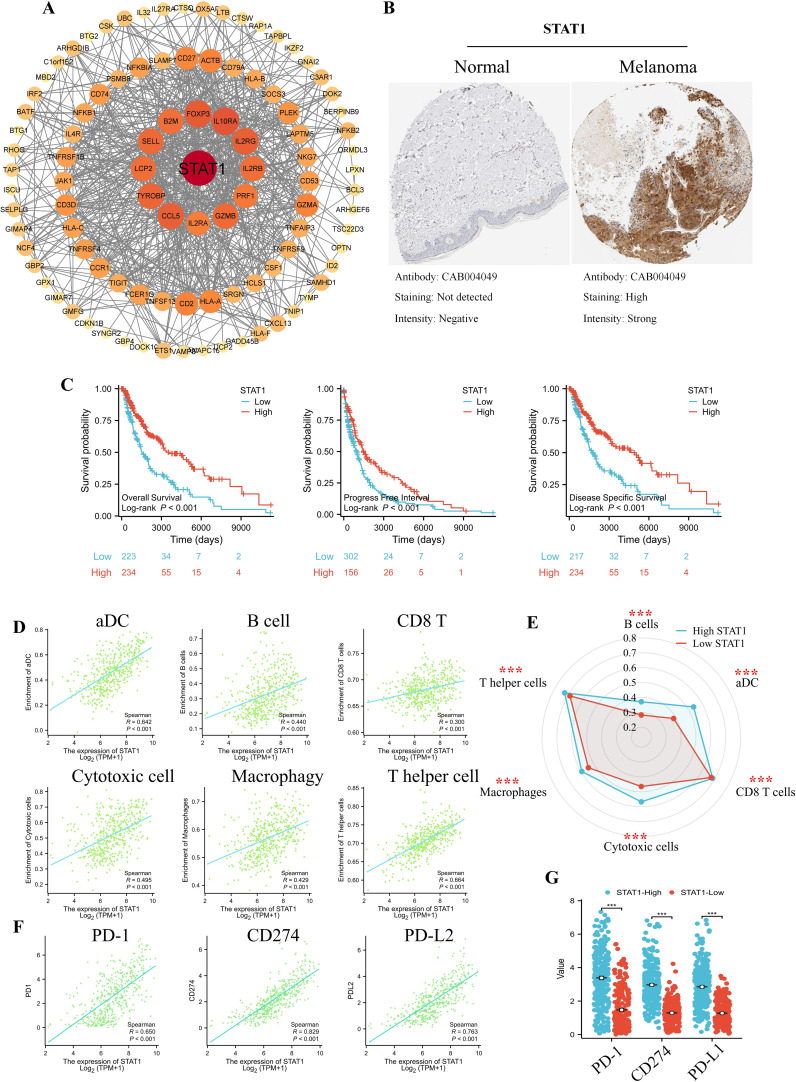
STAT1 is a potential target gene. **(A)** Hub genes STAT1 in the protein-protein interaction (PPI) network was identified using Cytoscape. **(B)** The protein expression levels of STAT1 in Human Protein Atlas (HPA) database based on immunohistochemistry analysis. The left side of the image represents normal skin tissue, which exhibits minimal STAT1 expression, as expected in healthy controls. The right side of the image shows a melanoma sample. The lower part of the melanoma image, where STAT1 high expression is detected, corresponds to the tumor-infiltrating immune cells. **(C)** Kaplan-Meier survival curve of overall survival, progress free survival, and disease free survival between patients with a high and low STAT1 expression in the TCGA-SKCM patients. **(D)** Scatter plot and **(E)** immunogram radar plot showing the correlation between STAT1 and tumor infiltration cells, included aDC, B cell, CD8 T, Cytotoxic cell, Macrophage, and T helper cell. **(F)** Scatter plot and **(G)** box plot showing the correlation between STAT1 and immune checkpoint, included PD-1, PD-L1, and PD-L2 in the TCGA dataset. ns indicates no significant difference; *P < 0. 05, **P < 0. 01; ***P < 0. 001.

Among the 10 Treg.Sig genes, the crucial one STAT1 was predicted through PPI network, which was selected for further analysis (**Figure 3A**). The protein expression levels of STAT1 in SKCM were observed by analyzing the immunohistochemical staining images in the HPA database. The levels of STAT1 expression were found to be elevated in SKCM tissue compared to normal skin tissue, as shown in **Figure 3B**. Notably, patients harboring higher STAT1 status had better survival than those with a lower STAT1 status (**Figure 3C**). In order to delve deeper into the underlying mechanism, we examined the connection between STAT1 and tumor-infiltrating cells. Our analysis revealed a positive correlation between STAT1 and the infiltration of immune cells that target tumors, such as aDC, B cells, CD8 T cells, cytotoxic cells, and macrophage cells (**Figures 3D, E**). Adding further complexity, an analysis was conducted on immune checkpoint proteins such as PD-1, PD-L1, and PD-L2. Likewise, there was a strong connection found between STAT1 and the primary immune checkpoint markers (PD-1 R=0.650, P<0.001; PD-L1 R=0.829, P<0.001; and PD-L2 R=0.763, P<0.001) (**Figures 3F, G**). These results indicated the genetic and expression alteration landscape of STAT1 in melanoma, suggesting dysregulating STAT1 participates in tumor immune microenvironment in melanoma contexts.

### STAT1 mutation analysis in melanoma

**Figure 4:**
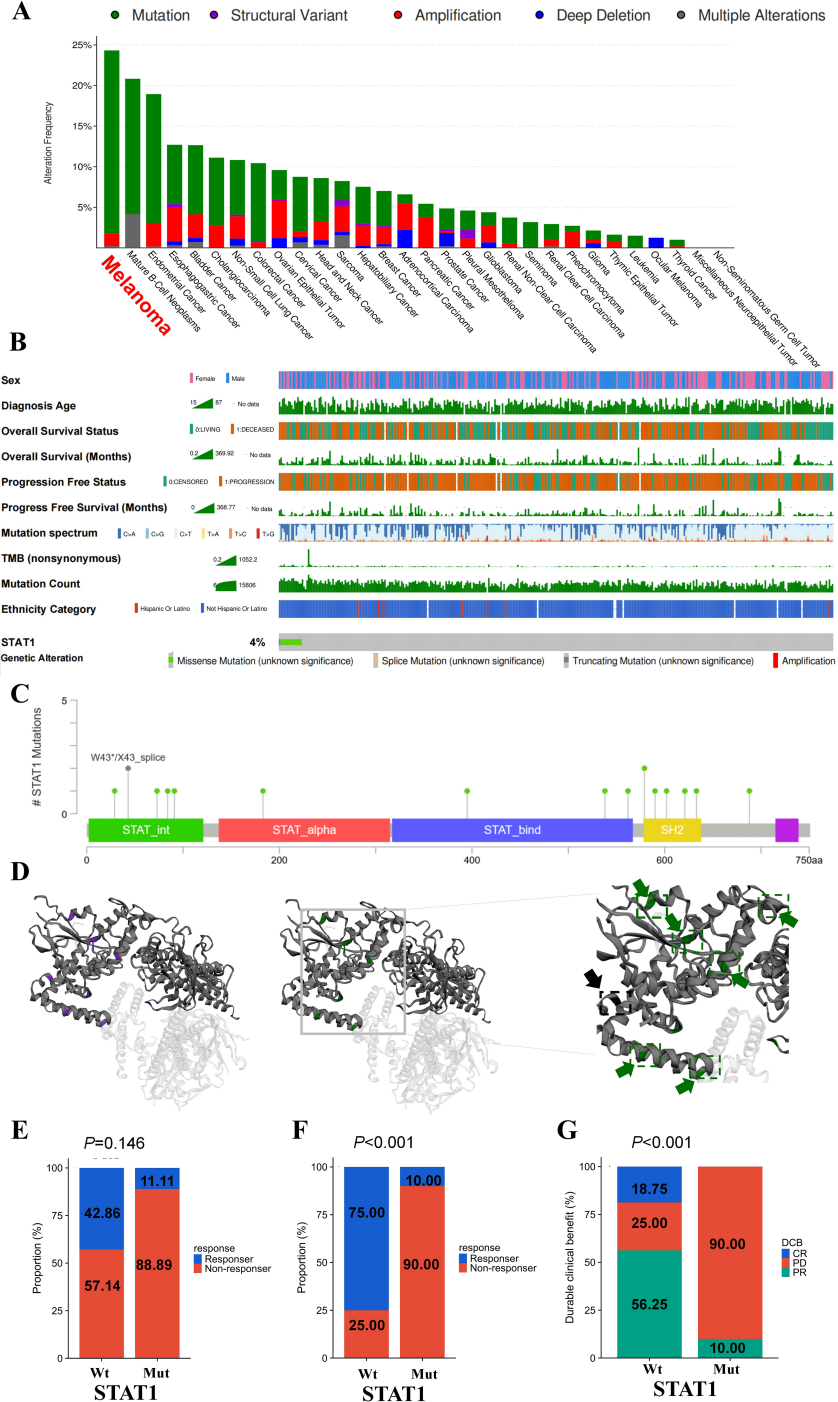
Association of STAT1 mutations with melanoma ICIs outcomes. **(A)** Mutational landscape of STAT1 across 30 cancers. **(B)** Association of STAT1 mutation and clinical characteristics in melanoma cohorts. Lollipop plot **(C)** and 3D landscape **(D)** showed the mutated structure of STAT1 in melanoma. **(E)** Proportion of responders to ICIs in melanoma patients with FGFR mutations versus FGFR wild-type. Comparison of the proportion of treatment response **(E, F)** and durable clinical benefit (complete response (CR), partial response (PR), stable disease (SD) and progression disease (PD)) **(G)** between STAT1 Mut and STAT1 Wt in melanoma ICI-cohort.

Subsequently, we explored weather STAT1 mutation was correlated with ICI treatment response. Initially, we confirmed the mutation rate of STAT in different types of cancers. Melanoma ranked 1st with a mutation frequency of 24.32% among all 30 cancers, as depicted in (**Figure 4A**), followed by mature B-Cell neoplasms and Endometrial Carcinoma. Subgroup analyses revealed that the occurrence rate of STAT1 mutations in melanoma was 3.87% (**Supplementary Figure S11A**). Various clinical features of individuals in the TCGA cohort, such as age, sex, survival rate, and tumor mutation burden, were analyzed and are depicted in **Figure 4B**. The Lollipop plot illustrates a total of 15 missense mutations (green) and 1 truncating (black) mutation in 750 amino acids long STAT1 protein (**Figures 4C, D**). Importantly, there was a correlation between STAT1 mutation and response to ICI treatment, showing a higher response rate in patients with STAT1-Wt compared to those with STAT1-Mut (**Figures 4E, F**). In the meantime, the STAT1-Wt group exhibited higher rates of complete response (CR) and partial response (PR), while the STAT1-Mut group had a higher rate of progression disease (PD) as shown in **Figure 4G**.

### Blocking STAT1 leads to ICIs resistance and defective N1 neutrophil polarization in B16-F10 melanoma mice receiving anti-PD-1

**Figure 5:**
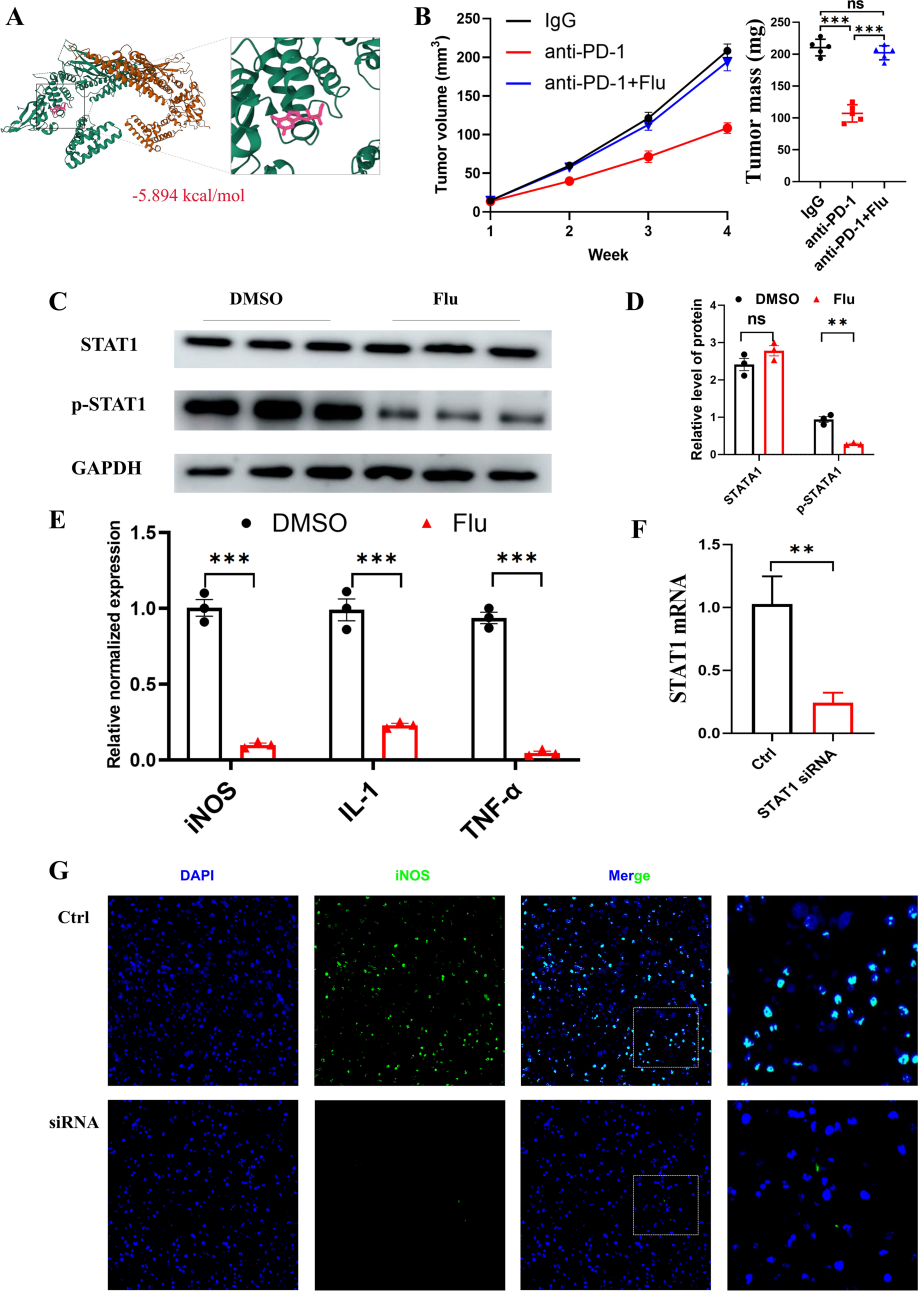
Inhibiting STAT1 impedes neutrophils polarization to the N1 phenotype. **(A)** The docking results of STAT1 with Fludarabine. **(B)** anti-PD-1 (200 µg per mouse), IgG control, or anti-PD-1 plus Fludarabine were intraperitoneally injected into mice once a week beginning on day 7 after B16-F10 cells were subcutaneously implanted. Tumors were measured once a week for 4 weeks. The tumor growth curves are shown in the left panel. Tumor weight at week 4 shown on right panel. **(C, D)** Western blot results of phosphorylation of STAT1 and total STAT1 expressions in macrophages neutrophils incubated with Fludarabine or DMSO. **(E)** Relative mRNA expression of N1 markers (iNOS, IL‐1, TNF‐α) in response to Fludarabine in N1 neutrophils (qRT‐PCR, n=3). **(F)** Mouse primary neutrophils were treated with STAT1 siRNA and STAT1 expressions were examined by qRT-PCR. GAPDH was used as a normalization control (n=3). **(G)** Changes in the expression of iNOS in N1 polarization by immunofluorescence. ns indicates no significant difference; *P < 0. 05, **P < 0. 01; ***P < 0. 001.

The anticancer role of STAT1 is thought to be essential in the process of tumorigenesis, where it fosters the disruption of transformed cells by the immune cells (29). Thus, we assessed the effect of STAT1 blockage on anti-PD-1 efficacy in the context of B16-F10 melanoma-bearing mice, thereby elucidating whether STAT1 is associated with immunotherapy resistance. We first performed a molecular docking analysis to assess the affinity of Flu for its target, STAT1. The findings indicated that Flu attaches to STAT1 by forming observable hydrogen bonds and robust electrostatic interactions, resulting in a binding energy of -5.894 kcal/mol, suggesting a very stable attachment (**Figure 5A**). Significantly, we noticed increased suppression of tumor growth when using anti-PD-1 therapy (**Figure 5B**). Nevertheless, tumors in the Flu treatment group eventually acquired adaptive resistance to the anti-PD-1 treatment, reaching a primary tumor size comparable to the controls by the fourth week (**Figure 5B**). We isolated neutrophils from harvested tumor tissue by using MACS neutrophil isolation kit, and analyzed whether cell phenotype of neutrophils is affected after mice treated with Flu. As illustrated in **Figures 5C, D**, STAT1 phosphorylation of neutrophils in B16-F10 tumor tissues was blocked in mice treated with Flu. Strikingly, blockade of STAT1 by Flu reversed N1 polarization-induced elevation of iNOS, IL-1, and TNF-α mRNA levels in neutrophils (**Figure 5E**). Furthermore, in order to confirm these findings, we utilized siRNA technology to knock down the expression of STAT1. The results suggested that STAT1 siRNA significantly down-regulated the expression of STAT1 (**Figure 5F**), accompanied by immunofluorescence showing the expression of N1-tagged iNOS was also reduced (**Figure 5G**).

### Blocking STAT1 shifts more neutrophils to N2 subtype and promotes tumor progression in B16-F10 melanoma mice receiving anti-PD-1

**Figure 6:**
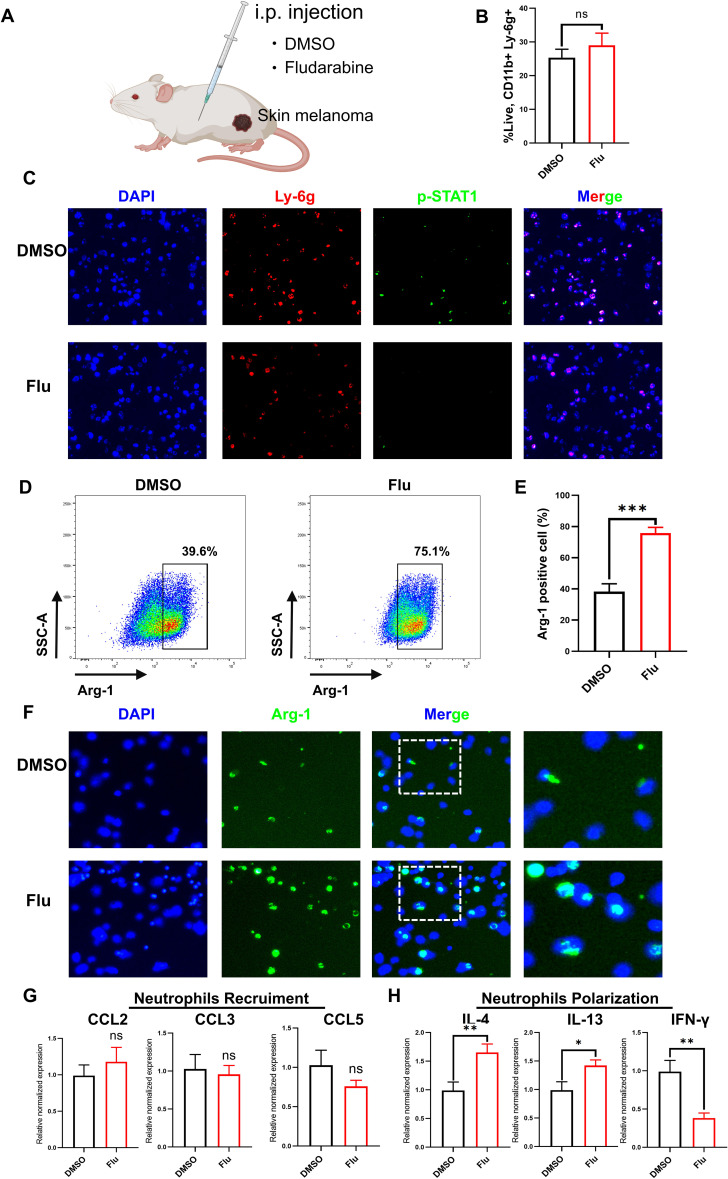
Melanoma TME converts more neutrophils to the N2 phenotype when STAT1 is inhibited. **(A)** Schematic diagram of the experimental procedure showing the injection of DMSO or Fludarabine into mice bearing B16-F10 melanoma allografts. **(B)** Flow cytometry analyzed the proportion of total neutrophils (CD11b+Ly-6G+). **(C)** Representative immunofluorescence images of Ly6G+pSTAT1+ neutrophils in B16-F10 melanoma allografts from DMSO or Fludarabine treated mice. **(D)** Flow cytometry analyzed the proportion of N2 phenotype neutrophils (Ly-6G+Arg1+). **(E)** Quantitative analysis of the results in **(D)**. n=3. **(F)** Co-culturing DMSO- or Fludarabine-treated neutrophils with B16-F10 melanoma cells, followed by immunofluorescence detection of N2 (Arg-1+) neutrophils. **(G)** Expressions of chemokine CCL2, CCL3, and CCL5 in B16-F10 melanoma allografts were examined by qRT-PCR. GAPDH was used as a normalization control (n=3). **(H)** Expressions of cytokines IL-4, IL-13, and IFN-gamma in B16-F10 melanoma allografts were examined by qRT-PCR. GAPDH was used as a normalization control (n=3). ns indicates no significant difference; *P < 0. 05, **P < 0. 01; ***P < 0. 001.

The above results which showing patients/mice with STAT1 mutation/inhibition harboring lower response rate during ICI treatment promote us to explore the underlying mechanisms of resistance. We suspected that malfunction of STAT1 may increase the susceptibility of neutrophils switching to a protumor suntype in melanoma tumor microenvironment (TME). We first validated whether STAT1 inhibition increase the infiltration of neutrophils. As shown in **Figures 6A, B**, during anti-PD-1 therapy, treatment of Flu in B16-F10 bearing mice did not alter the proportion of CD11b+Ly-6G+ neutrophils, indicating that STAT1 inhibition did not affect neutrophils infiltration into tumor tissue. As expect, treatment of Flu decreased the phosphorylation of STAT1 in neutrophils in B16-F10 melanoma tissue (**Figure 6C**). Surprisingly, flow cytometry analysis showed that treatment of Flu in B16-F10 melanoma bearing mice significantly promoted the phenotype switch of neutrophils toward a N2 subtype (Arg-1+) (**Figures 6D, E**). These results indicated that melanoma TME could skew more neutrophis to N2 subtype when STAT1 was inhibited. To further validated this, we used cell-cell coculture system to explore weather B16-F10 melanoma cells could drive more neutrophils toward N2 subtype *in vitro*. We isolated neutrophils from bone marrow and detected the expression of N2 marker after co-culture. The results showed neutrophils expressed Arg-1 after cocultured with B16-F10 melanoma cells, and this trend was intensified while STAT1 was inhibited by Flu (**Figure 6F**). Further investigations were conducted into the tissue-specific chemokine and cytokine profile influencing neutrophil recruitment and polarization. It was discerned that within Flu treated B16-F10 melanoma sites, there were no alteration in the levels of CCL2, CCL3, and CCL5, which are pivotal in mediating neutrophil recruitment (**Figure 6G**). Nevertheless, there were noticeable increased in the concentration of IL-4 and IL-13, cytokines crucial for the polarization towards an N2 neutrophil phenotype, whereas the levels of IFN-gamma, a cytokine essential for N1 neutrophil programming, were decreases (**Figure 6H**).

**Figure 7:**
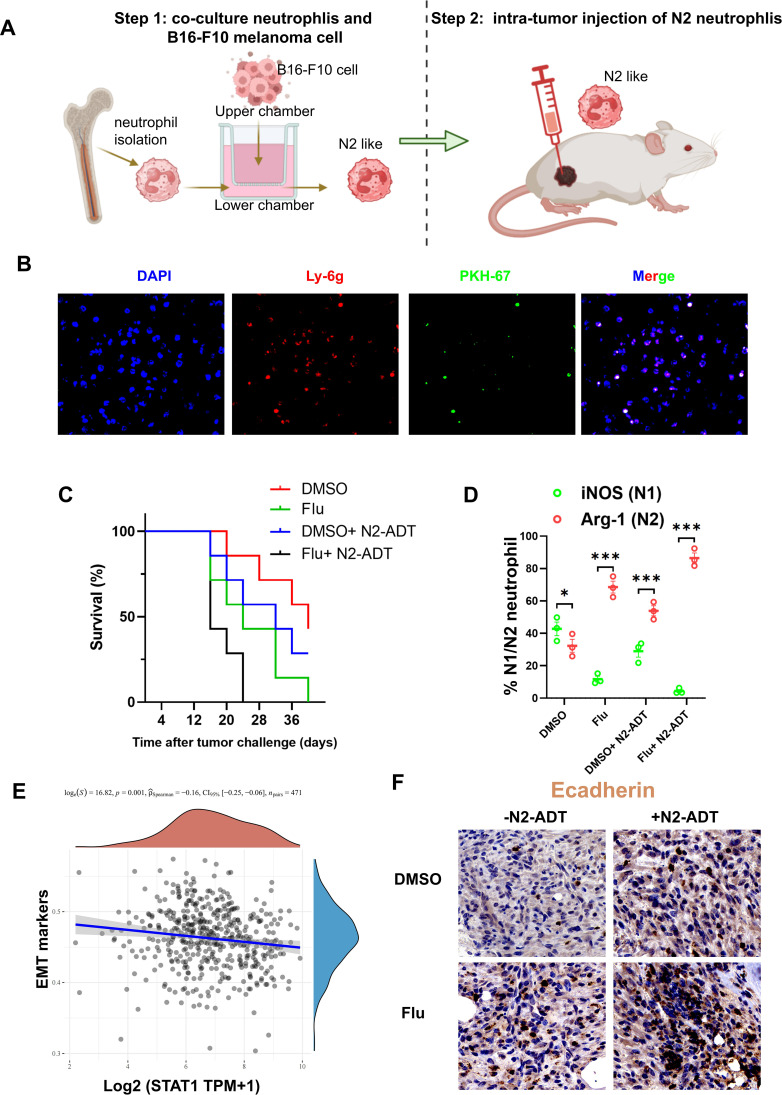
Inhibition of STAT1 induces more N2 neutrophils and thus promotes B16-F10 melanoma growth. **(A)** Schematic diagram of the experimental procedure showing the induction of N2 neutrophils (step1) and adoptive transfer of N2 neutrophils into mice bearing B16-F10 melanoma (step2). **(B)** Immunofluorescence showing the injection of PHK67-labeled N2 neutrophils into B16-F10 melanoma allografts. **(C)** Quantification of N1 (iNOS+) and N2 (Arg-1+) neutrophils for the panel. **(D)** Overall survival in mice implanted with B16-F10 melanoma allografts and further treated with DMSO, Fludarabine, or adoptive transfer (ADT) of N2 neutrophils. **(E)** Scatter plot revealing the correlation between STAT1 and epithelial to mesenchymal transitions (EMT). **(F)** Representative IHC images for E-cadherin in B16-F10 melanoma allografts derived from the experiment in **(D)**. ns indicates no significant difference; *P < 0. 05, **P < 0. 01; ***P < 0. 001.

In order to study the immediate impact of these N2-neutrophils on tumor progression *in vivo*, we conducted the transfer of N2-neutrophils (Ly-6G+Arg-1+), which were extracted from the bone marrow of healthy mice without tumors and then co-cultured with B16-F10 melanoma cells (**Figure 7A**). We employed fluorescent tracer technology to track intra-tumor injected N2 neutrophils. As shown in **Figure 7B**, PKH67-labeled N2 neutrophils remained in the tumor tissue 1 day post injection. Surprisingly, in the context of anti-PD-1 treatment, administering Flu to mice with B16-F10 melanoma notably reduced their OS compared to the control group (**Figure 7C**). Furthermore, the decline in OS was exacerbated by the introduction of N2-neutrophils through adoptive transfer (**Figure 7C**). Flow cytometry analysis was used to examine the expression of pertinent N1 and N2-neutrophil markers in B16-F10 melanoma allografts. In our study, we observed that both Flu and adoptive transfer of N2-neutrophils led to a rise in N2-neutrophil (Arg-1+) levels in B16-F10 melanoma allografts when compared to the DMSO control group, which predominantly showed N1- neutrophil infiltration (iNOS) (**Figure 7D**). To figure out the underlying mechanisms that STAT1 regulated melanoma resistance, we analyzed the correlation between STAT1 and epithelial-mesenchymal transition (EMT). As an important mechanism by which cancer cells obtain highly invasive phenotype, EMT is a process acquiring mesenchymal features in epithelial cells. It was demonstrated that immune cells within the tumor microenvironment play a crucial role in influencing this plasticity of tumor cells (30). Correlation analyses were conducted to examine the impact of STAT1 on these processes, revealing a negative correlation between STAT1 and EMT markers (**Figure 7E**). To verify this result, we examined the expression of epithelial (E-cadherin) marker by immunohistochemical staining. As shown in **Figure 7F**, both Flu and adoptive transfer of N2-neutrophils resulted in elevated levels of E-cadherin expression.

### The N2 subtype neutrophils induced by B16-F10 melanoma cells can in turn promote melanoma cell invasion, proliferation, and epithelial-mesenchymal transition

**Figure 8:**
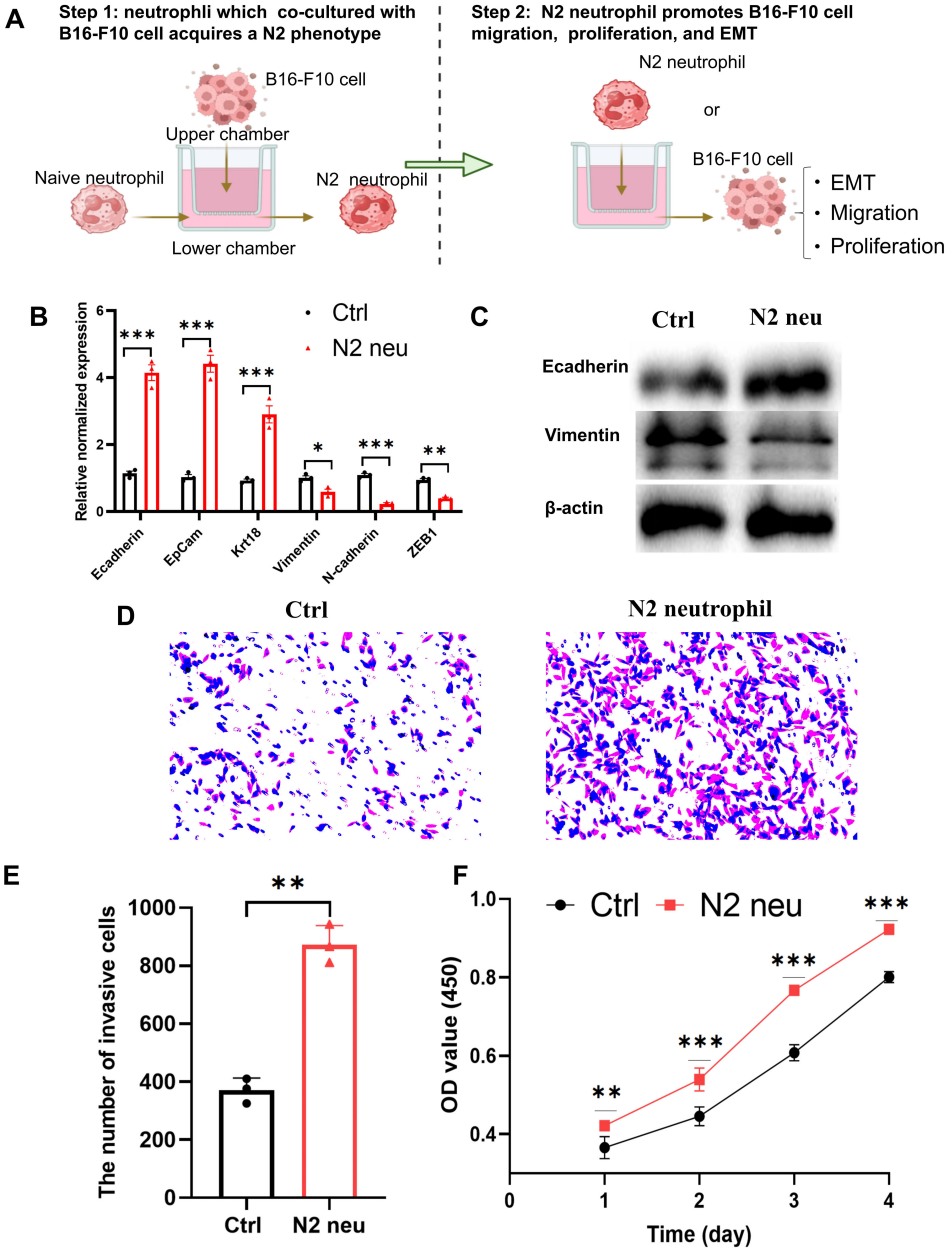
N2 neutrophils promotes B16-F10 EMT, invasion and proliferation. **(A)** Schematic diagram of the experimental procedure showing the induction of N2 neutrophils (step1) and N2 neutrophils promote B16-F10 melanoma EMT, invasion, and proliferation (step2). **(B)** B16-F10 melanoma cells were co-cultured with the N2 neutrophils. Cells were then examined for the expression of epithelial (E-cadherin, EpCam, Krt18) and mesenchymal (Vimentin, N-cadherin, ZEB1) markers (qRT‐PCR, n=3). **(C)** B16-F10 melanoma cells were co-cultured with the N2 neutrophils. Cells were then examined for the expression of epithelial (E-cadherin) and mesenchymal (Vimentin) markers by western blot. **(D)** Transwell assays were carried out to evaluate the invasive potentials of B16-F10 melanoma cells. **(E)** Quantification of invasive cell number in transwell assay. **(F)** CCK-8 assays were carried out to evaluate the proliferative potentials of B16-F10 melanoma cells. ns indicated no significant difference; *P < 0. 05, **P < 0. 01; ***P < 0. 001.

Based on the aforementioned *in vivo* findings, we conducted *in vitro* studies to examine how N2 neutrophils impact melanoma EMT, invasion, and proliferation. In order to achieve this goal, melanoma B16-F10 cells were cultured with N2 neutrophils (**Figure 8A**). As shown in **Figures 8B**, **C**, B16-F10 cells co-cultured with N2 neutrophils showed higher epithelial markers (Ecadherin, EpCam, Krt18) expression but lower mesenchymal markers (Vimentin, N-cadherin, ZEB1). Additionally, N2 neutrophils increased the invasion of B16-F10 cells as shown in transwell assays (**Figures 8D, E**). Likewise, in CCK-8 experiments, it was noted that the presence of N2 neutrophils led to a notable enhancement in the proliferation ability of B16-F10 melanoma cells as opposed to the control group (**Figure 8F**). Collectively, these findings indicate that the absence of STAT1 is associated with the pro-tumor activity of N2 neutrophils and resistance to melanoma immune checkpoint inhibitors.

## Discussion

Advancements in cancer immunotherapy have led to the ongoing discovery of predictive biomarkers for response to immunotherapy. Extensive research has been conducted on how the tumor microenvironment (TME) affects the effectiveness of cancer immunotherapy, with a particular emphasis on biomarkers associated with TME (31, 32). Nevertheless, there is a scarcity of reliable biomarkers derived from the TME of tumorigenesis to predict the response to immunotherapy and prognosis in SKCM. Advancements in single-cell RNA sequencing techniques have made it easier to analyze the molecular features of immune cells that have invaded tumors in the TME (33–35). This study utilized machine learning to analyze scRNA-seq and bulk-seq data, revealing ten Tregs marker genes showing significant expression changes in SKCM tissue. A novel prognostic Treg.Sig was further constructed based on the Tregs marker genes. An independent risk factor for SKCM patients was identified in the form of this Treg.Sig. Then, the prognostic performance of Treg.Sig was validated, revealing that Treg.Sig held predictive power even beyond other established signatures. Examination of biological pathways showed that genes associated with Treg.Sig were significantly present in immune profiles, with unique proportions of immune cells and immune-suppressive conditions identifying patients with a high-risk Treg.Sig score. Moreover, a low Treg.Sig risk score was found to be correlated with various chemotherapy and immunotherapy markers, suggesting that low-risk individuals may benefit more from ICI treatment. The pivotal gene STAT1 was confirmed to demonstrate its essential function in tumor immunogenicity. The research indicated that Treg.Sig could be a valuable predictor for prognosis and response to immunotherapy in patients with SKCM.

The Treg.Sig had ten Treg cell marker genes, including GBP2, RAP1A, BTG1, IL27RA, STAT1, TXNDC11, GBP4, ARID5A, CLEC2B, and SEL1L3. In the signature model, some genes presented protective or negative functions on the prognosis of SKCM patients. For example, an important positive relationship was observed between GBP2 and the infiltration of CD8+ T cells (36). GBP2, a member of the GTP superfamily, suppresses mitochondrial fission and cell invasion in various types of tumors. Herein, there was a favorable response in melanoma patients with higher GBP2 expression than those with low GBP2 expression (37). In addition, several cancer types involve EPAC/cAMP-RAP1A signaling axis in cell proliferation, differentiation, and cell-cell junction (38). Decreased survival was observed in primary melanoma cells following RAP1A knockdown, while metastatic melanoma cells showed increased proliferation rates (39). These data indicate that RAP1A plays a two-fold function in the survival and growth of melanoma cells, serving as a key factor in determining how primary and metastatic melanoma cells react to cAMP (39). As for the STAT1, it has previously been reported to be a tumor suppressor in hepatocellular carcinoma (40) and in esophageal squamous cell carcinoma (41). Melanoma research has shown that the eIF4F-STAT1-PD-L1 axis plays a role in controlling tumor immune evasion (42). In the present study, STAT1 was found to be associated with patient prognosis and tumor immunity. Mechanically, we identified that STAT1 was associated with tumor resistance and N2 neutrophil polarization during ICIs treatment. Therefore, the rise in STAT1 protein levels supports its potential as an anticancer agent in SKCM, suggesting that the genes identified in this research could be valuable targets for further investigation in the lab to uncover the molecular mechanisms behind SKCM resistance.

We validated the predictive performance of our Treg.Sig by comparing it to 51 previously published melanoma-related signatures in terms of 1-year, 3-year, and 5-year overall survival rates. The Treg.Sig we created stands out for its strong ability to predict prognosis and could be very useful in the future. The performance of the Treg.Sig was further validated in different clinical subgroups. Our signature was found to show statistically significant overall survival stratification for SKCM patients across all clinicopathologic subgroups. The Treg.Sig had strong predictive capability prompting investigations to determine its potential underlying mechanism of action. A total of 438 positively related genes and 28 negatively related genes strongly correlated with Treg.Sig were determined using correlation analysis. The GSEA analysis revealed that the associated genes were predominantly enriched in Treg cell-related biological processes, including allograft rejection, autoimmune thyroid disease, leishmaniasis, systemic lupus erythematosus, and type I diabetes mellitus. The enrichment of Treg-related pathways (allograft rejection, SLE, type I diabetes) aligns with recent findings that tumor-educated Tregs co-opt autoimmune-associated transcriptional programs to suppress antitumor immunity (43, 44). This molecular mimicry could explain why high Treg.Sig tumors exhibit both autoimmune-like inflammation and functional immunosuppression. Furthermore, we examined the discrepancy in immune cell infiltration between high- and low-risk SKCM patients, taking into account the influence of the tumor microenvironment on tumor prognosis. High-risk SKCM patients exhibited a reduced percentage of CD8+ T cells and NK cells, indicating an immunosuppressive tumor microenvironment. Part of the explanation for this could be that Tregs are able to directly suppress not just CD4+ helper T cells and CD8+ cytotoxic T cells, but also NK cells after cancer treatment (45). Additionally, Tregs and cancer cells work together to remodel the extracellular matrix, creating a barrier that prevents tumoricidal immune cells from penetrating solid tumors and delivering anticancer agents (45). Interestingly, the pRRophetic algorithm indicated that SKCM patients with low-Treg.Sig were more responsive to traditional chemotherapy drugs with diverse mechanisms of action—including the VEGFR inhibitor Pazopanib, microtubule depolymerizer Paclitaxel, and multi-targeted kinase inhibitor Tivozanib—compared to high-Treg.Sig patients. Prior studies suggest these drugs exert immunomodulatory effects beyond their canonical mechanisms. For instance, Pazopanib reduces immunosuppressive Tregs and myeloid-derived suppressor cells (MDSCs) while upregulating PD-1 expression on cytotoxic T cells, thereby augmenting tumor cell killing capacity (46). While our data associate low-Treg.Sig with improved drug sensitivity, we emphasize that these agents likely act through pleiotropic mechanisms: Pazopanib combines anti-angiogenic effects with Treg suppression, whereas Paclitaxel disrupts microtubules while synergize with pre-existing cytotoxic T cell populations. Critically, the predictive value of Treg.Sig may reflect a permissive TME in low-Treg.Sig tumors, where reduced immunosuppression enables chemotherapy-induced immunostimulatory effects (e.g., antigen release, T cell priming) to dominate. Future studies combining Treg-specific depletion models with pharmacogenomic profiling are warranted to disentangle direct Treg modulation from broader immune contexture-driven responses.

The discrepancy in immune cell penetration and inflammatory functions among different risk groups prompted our investigation into the predictive potential of Treg.Sig for immunotherapy response. Prior research has indicated that the expression of PD-L1 can predict how patients will respond to inhibitors targeting PD-1/PD-L1 (47). Moreover, TIDE is a recently identified predictor for immunotherapy that has shown superior predictive capabilities when compared to other biomarkers or indicators (48). We examined if Treg.Sig could serve as a biomarker for immunotherapy response by studying its correlation with the mentioned biomarkers. The results revealed that Treg.Sig low-risk patients had significantly higher TIDE scores and IPS scores. Typically, a lower TIDE score or a higher IPS score indicates a more favorable reaction to immunotherapy (49). A high IPS signifies increased immunogenicity, while a high TIDE score indicates a greater chance of tumor immune evasion. This research found that low-risk tumors exhibited greater IPS/immunogenicity but also had higher TIDE scores than the high-risk group. As a result, the reliability of Treg.Sig as a predictor should be confirmed in additional immunotherapy datasets. By examining the predictive power of Treg.Sig in three immunotherapy groups (GSE161801, GSE120575, GSE189125), we found that patients with a low risk profile were more responsive to ICI treatment. Consistent with these results were the findings of immune cell infiltration, indicating that the influence of the quantity and effectiveness of tumor-infiltrating T cells on the response to immunotherapy is more significant than that tumor immunogenicity (50, 51). The robust discrimination of Treg.Sig and favorable predictive metrics position it as a clinically actionable tool. Integrating Treg.Sig with existing biomarkers could enable precision stratification – for instance, combining it with PD-L1 status may identify ‘double-negative’ patients (low Treg.Sig/PD-L1+) who derive maximal benefit from PD-1/CTLA-4 combination therapy.

The interplay between STAT1 signaling and neutrophil polarization toward the N2 phenotype presents a compelling avenue for cancer therapeutic exploration. Emerging evidence suggests that STAT1 activation in neutrophils drives pro-inflammatory N1 polarization, characterized by enhanced ROS production and pro-tumorigenic cytokine secretion (52, 53). Conversely, inhibition of STAT1 may skew neutrophils toward the N2 phenotype, which exhibits anti-inflammatory and tissue-repair properties, potentially attenuating tumor-associated inflammation and metastasis. For instance, subclinical LPS-induced activation of STAT1/STAT5 in neutrophils exacerbates inflammatory polarization, implying that targeted STAT1 suppression could reverse this phenotype (52).

Although this study yielded important results, several limitations were warrant cautious interpretation. First, the retrospective nature of public datasets introduces inherent selection biases – for example, TCGA-SKCM overrepresents treatment-naïve primary tumors (73%) compared to metastatic cases routinely seen in clinics. Second, technical variability in Treg quantification (e.g., xCELL vs. CIBERSORT algorithms) may confound immune infiltration analyses, though we mitigated this through multi-algorithm validation. Third, the selected genes were Tregs specific marker genes, yet the TME is highly spatially heterogeneous (54). Hence, the prognosis-prediction ability of the signature may be limited. Finally, the risk signature based on Tregs was created by analyzing historical data from publicly available databases. Therefore, it should be validated in further prospective and multi-center SKCM cohorts in the future.

## Conclusions

We offer the initial concrete clinical proof that a signature relying on Treg cells is linked to resistance to immunotherapy in patients with SKCM. By utilizing machine learning to merge single-cell and bulk RNA sequencing data, we created a gene expression profile called Treg.Sig that surpasses existing signatures in forecasting survival rates for patients with SKCM. Additional examination of genes within Treg.Sig uncovered several possible targets for therapy. The research introduces a hopeful approach to identifying individuals who may respond well to immunotherapy, and suggests potential solutions for overcoming resistance to ICI by focusing on Tregs marker genes to enhance the body’s ability to fight against tumors.

## Data Availability

The data presented in the study are deposited in the GEO repository, accession number GSE123139, GSE65904, GSE161801, GSE120575, and GSE189125.
