# Supplementary material for: Unveiling the power of Treg.Sig: a novel machine-learning derived signature for predicting ICI response in melanoma
Source: Front Immunol. 2025 Mar 28;16:1508638. doi: 10.3389/fimmu.2025.1508638 (PMC11985843; doi:10.3389/fimmu.2025.1508638)
Supplement: Supplementary file 1 [file DataSheet1.docx]

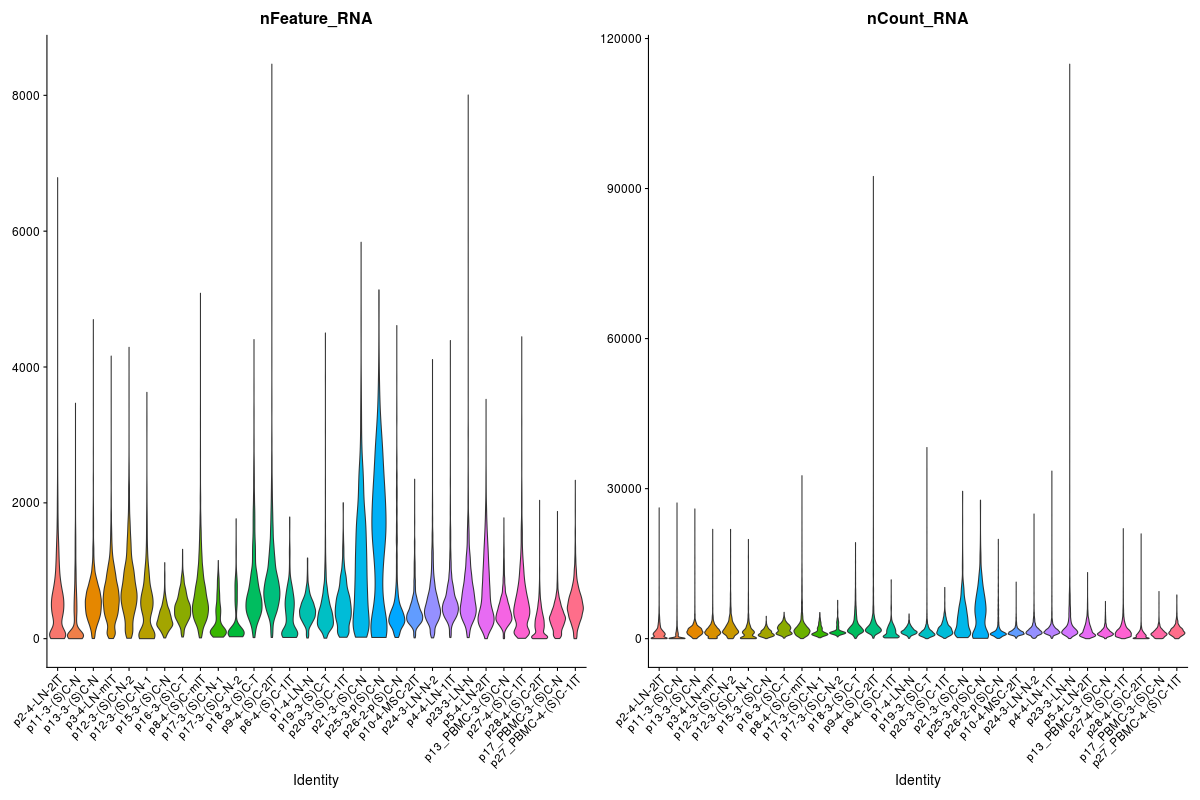


**Supplemental Fig. 1** **High-quality cell selection for Single-cell RNA-sequencing.**


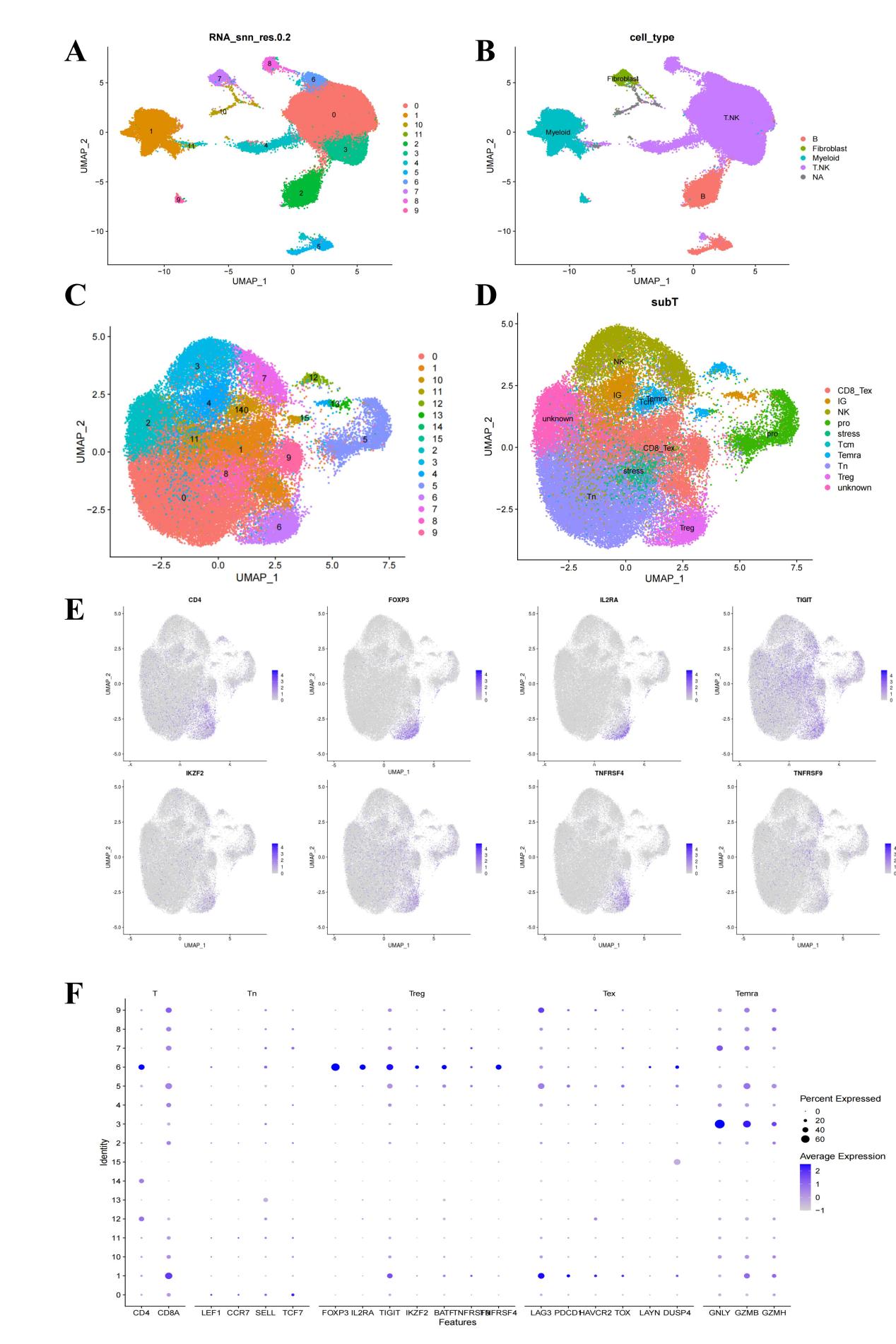
**Supplemental Fig. 2** **Identification of regulatory T cell marker genes by single-cell RNA-sequencing analysis. (A)** UMAP plot colored by various cell clusters (64,071 cells from 30 melanoma patients; Cells were clustered into 12 types via UMAP dimensionality reduction algorithm); **(B)** The cell types identified by marker genes. **(C)** 16 sub-clusters of T and NK cell. **(D)** Subgroups identified by marker genes in T and NK cell. **(E)** Expression profile of Treg marker gene: FOXP3, IL2RA, TIGIT, IKZF2, BATF, TNFRSF4, and TNFRSF9 in subgroups. **(F)** The bubble plots show the gene expression profiles of the marker gene in each subgroup. Cell clusters are listed on the Y-axis, and recognized marker genes are listed on the X-axis. The size of the dots reflects the percentage of expression of each gene in the cells of each cluster; the color of the dots represents the expression level.

**
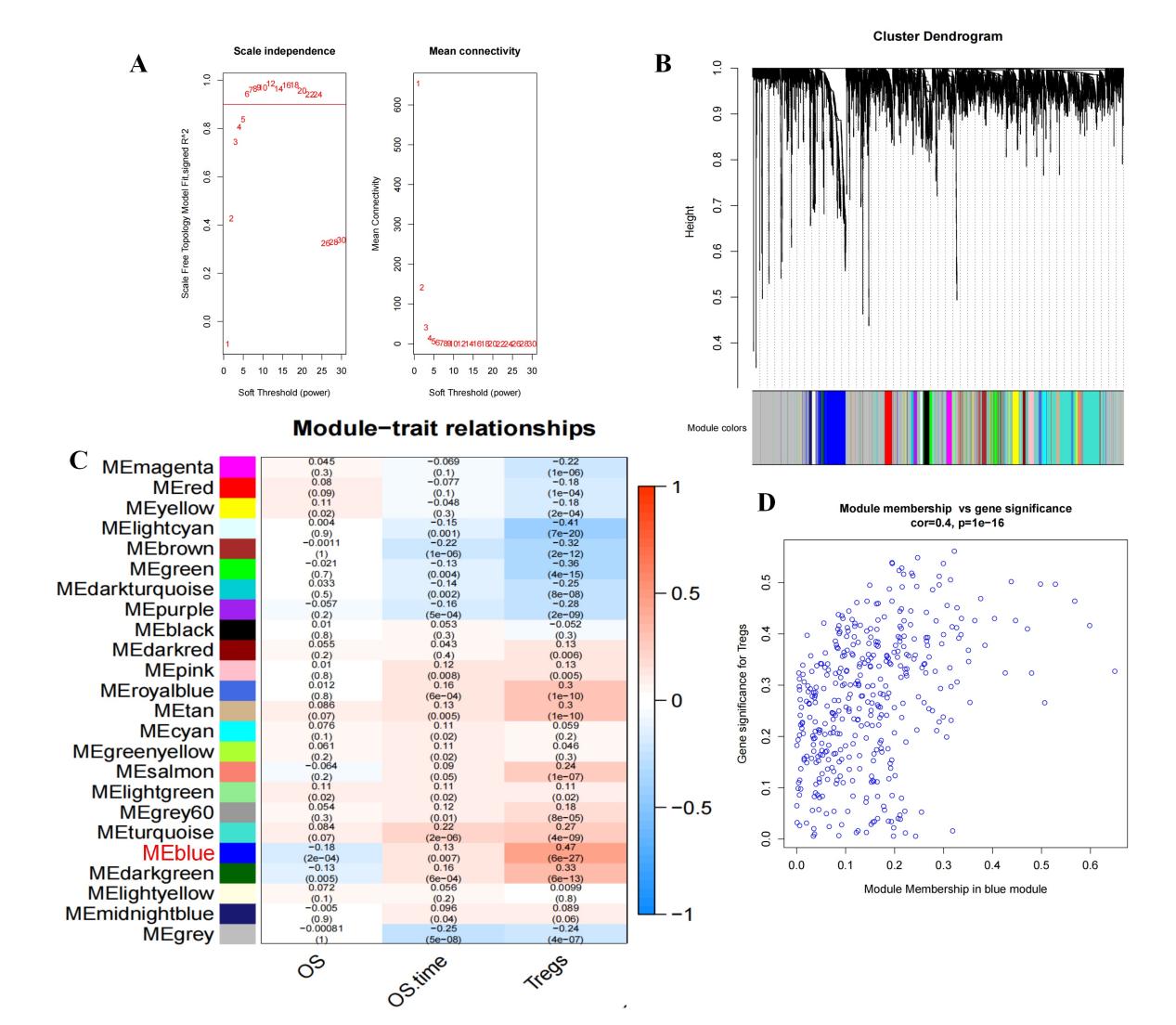
Supplemental Fig. 3 Identification of regulatory T cell marker genes by bulk RNA-sequencing analysis. (A)** Determination of soft-thresholding power in the weighted gene co-expression network analysis (WGCNA). **(B)** Dendrogram of all differentially expressed genes clustered based on a dissimilarity measure (1-TOM). **(C)** Correlations between co-expression gene modules and OS, OS time, and Tregs. Each cell contains the corresponding correlation and p-value. **(D)** Scatter plots of gene significance and module membership for genes in the blue modules.


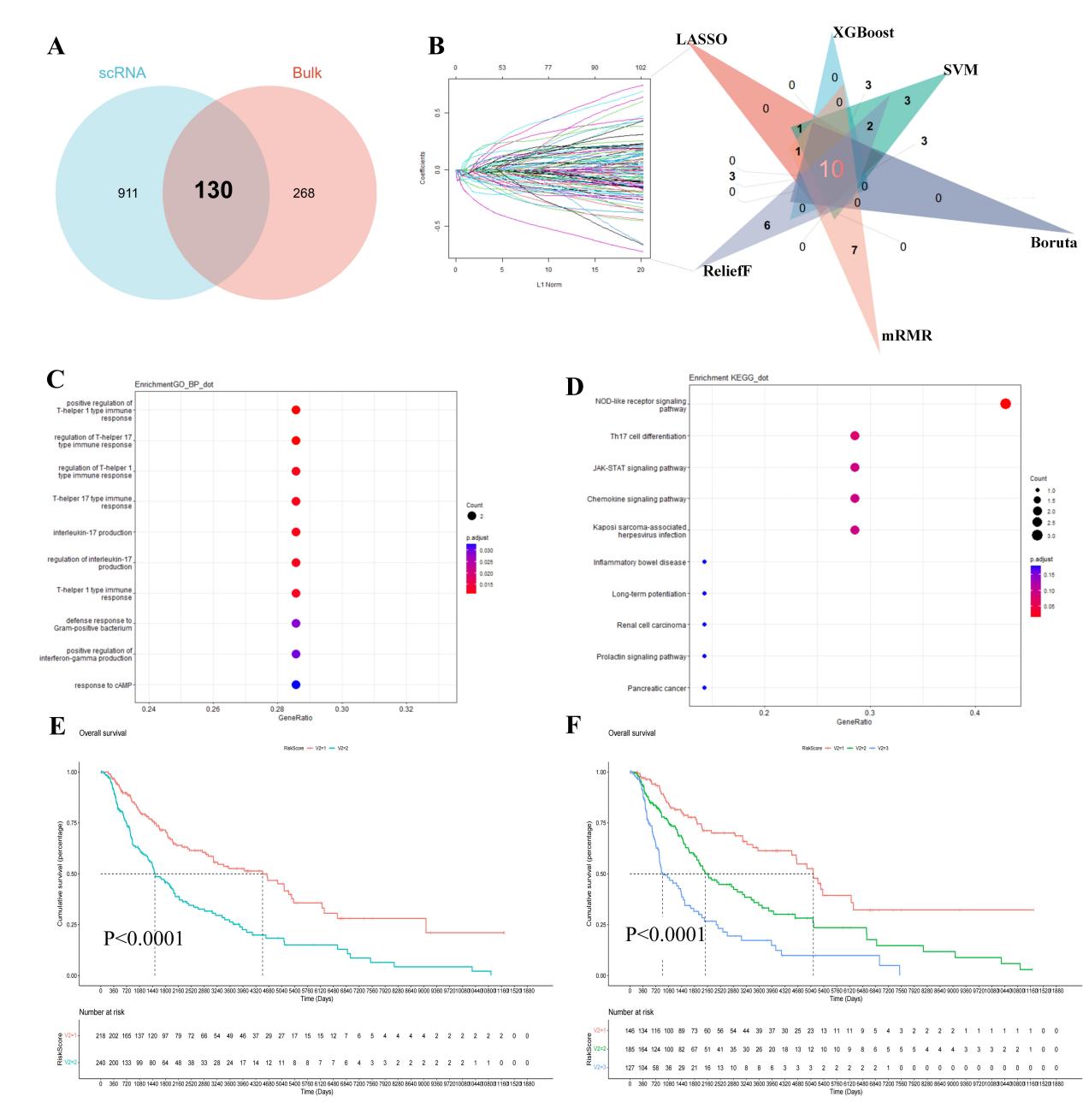


**Supplemental Fig. 4 Construction and validation of 10 hub marker genes for regulatory T cells (Tregs).** (**A**) Venn plot showing the intersected candidate genes based on scRNA-seq and bulk-seq. (**B**) Six machine learning algorithms identified 10 hub Tregs associated genes. (**C**) GO and (**D**) KEGG analyzed the reliability of 10 hub Tregs associated genes. Unsupervised machine learning (**E**: k values=2; **F**: k values=3) verified the prognosis value of 10 hub Tregs associated genes.


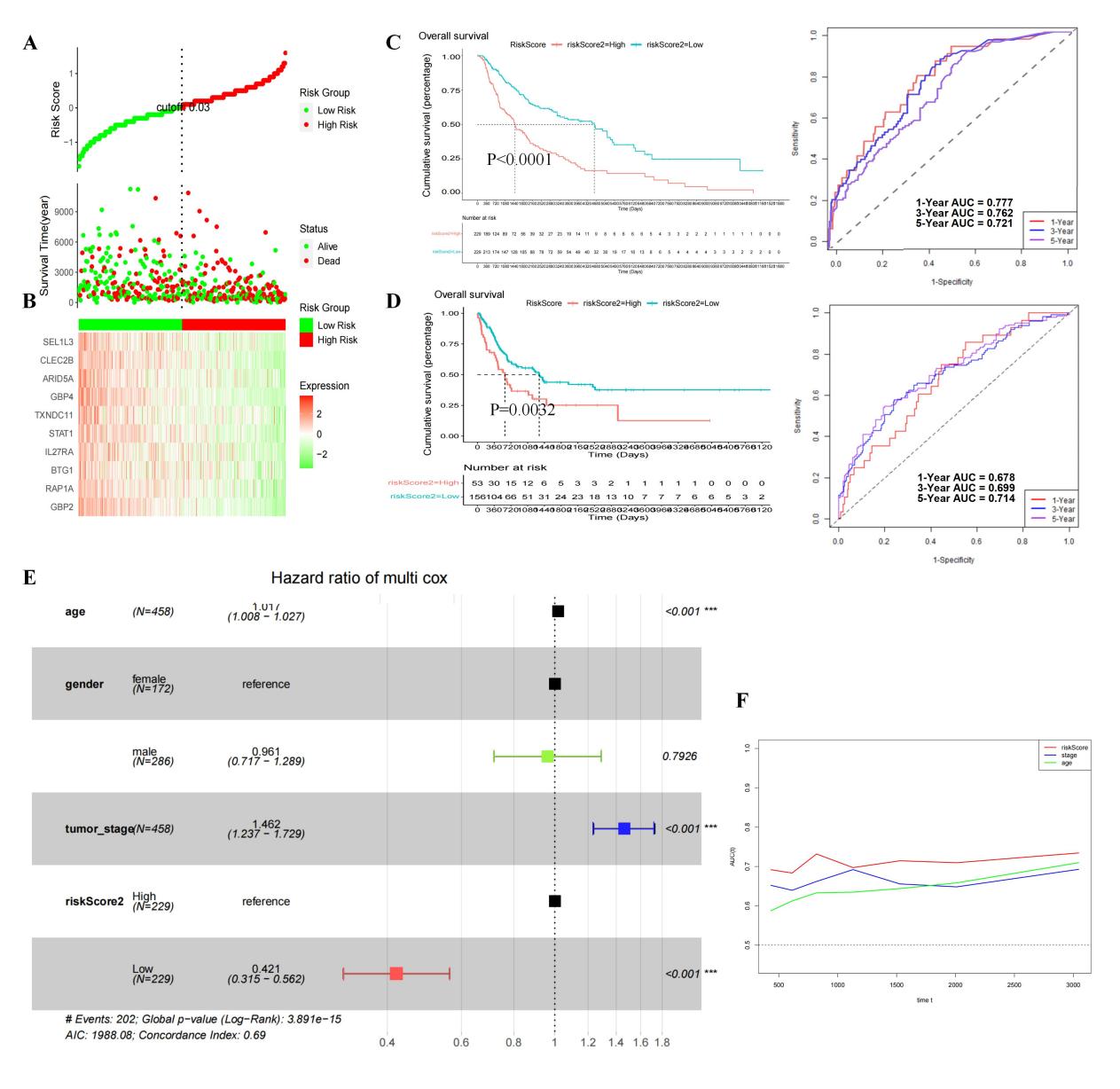


**Supplemental Fig. 5** **Validation of prognostic performance of Tregs signature (Treg.Sig) for overall survival (OS) in SKCM patients.** (**A**) The distribution of risk score and survival status. (**B**) Thermal maps of the 10 hub gene expression profiles in the high- and low-risk subgroups. The green curve denotes “low-risk” and the red curve means “high-risk”. The risk scores of SKCM patients in TCGA were calculated by the Tregs signature and divided into two subgroups according to the medium score. The survival curves and ROC curves based on the Tregs signature in the TCGA derivation cohort (**C**) and GSE65904 validation cohort (**D**). (**E**) Multivariate Cox analysis of clinical information and Tregs signature. (**F**) Time-dependent AUCs comparing the predictive value among Tregs signature, age, and tumor staging.


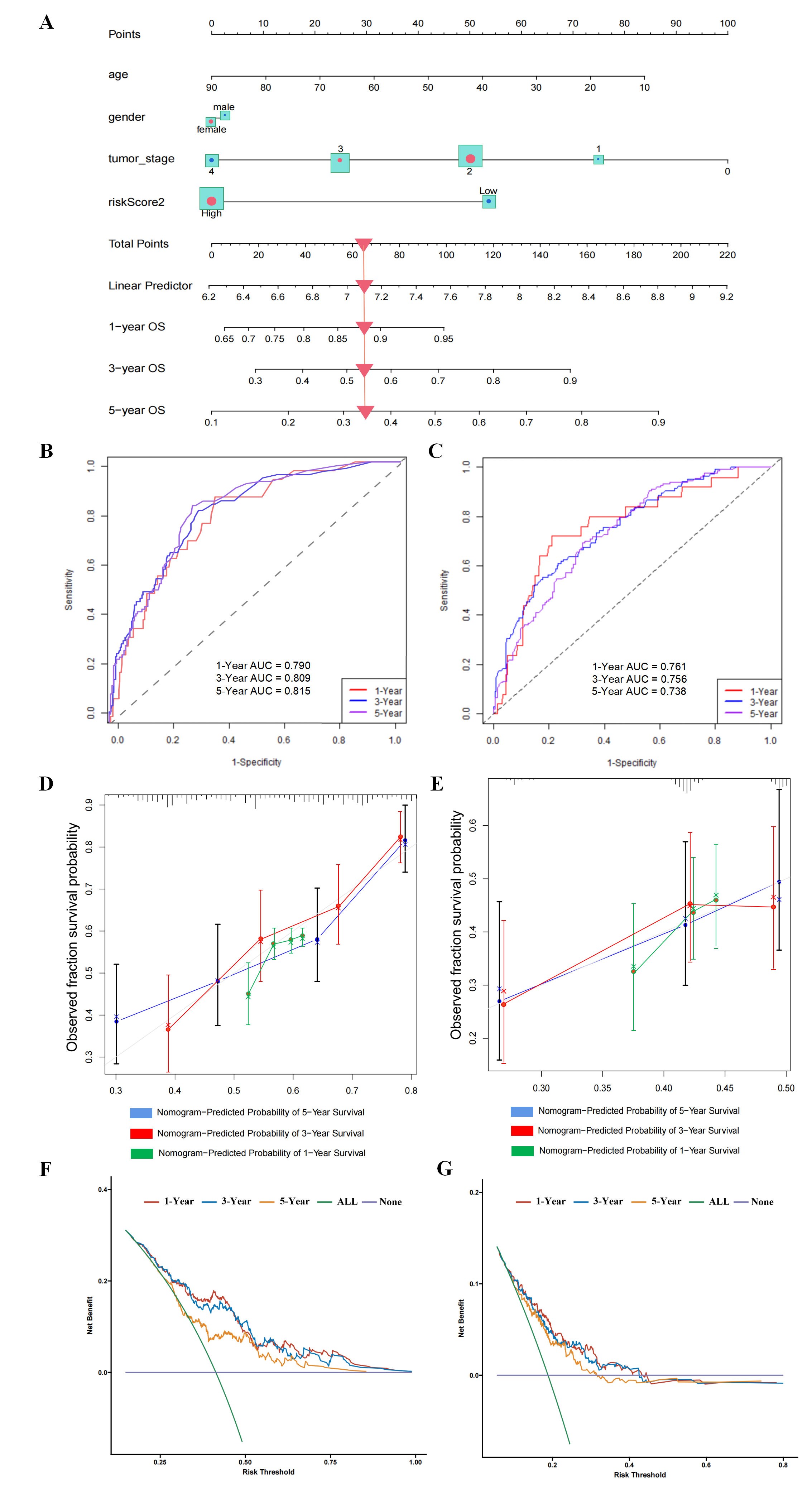


**Supplemental Fig. 6** **Construction and validation of a nomogram for predicting the prognosis of malignant melanoma patients.** (**A**) A nomogram based on RiskScore signature and related clinical information. ROC curves of the nomogram in TCGA cohort (**B**) and GEO65904 cohort (**C**) predicting 1-, 3-, and 5-year survival probabilities. Calibration of the nomogram in TCGA cohort (**D**) and GEO65904 cohort (**E**). DCA results of the nomogram in TCGA cohort (**F**) and GEO65904 cohort (**G**).


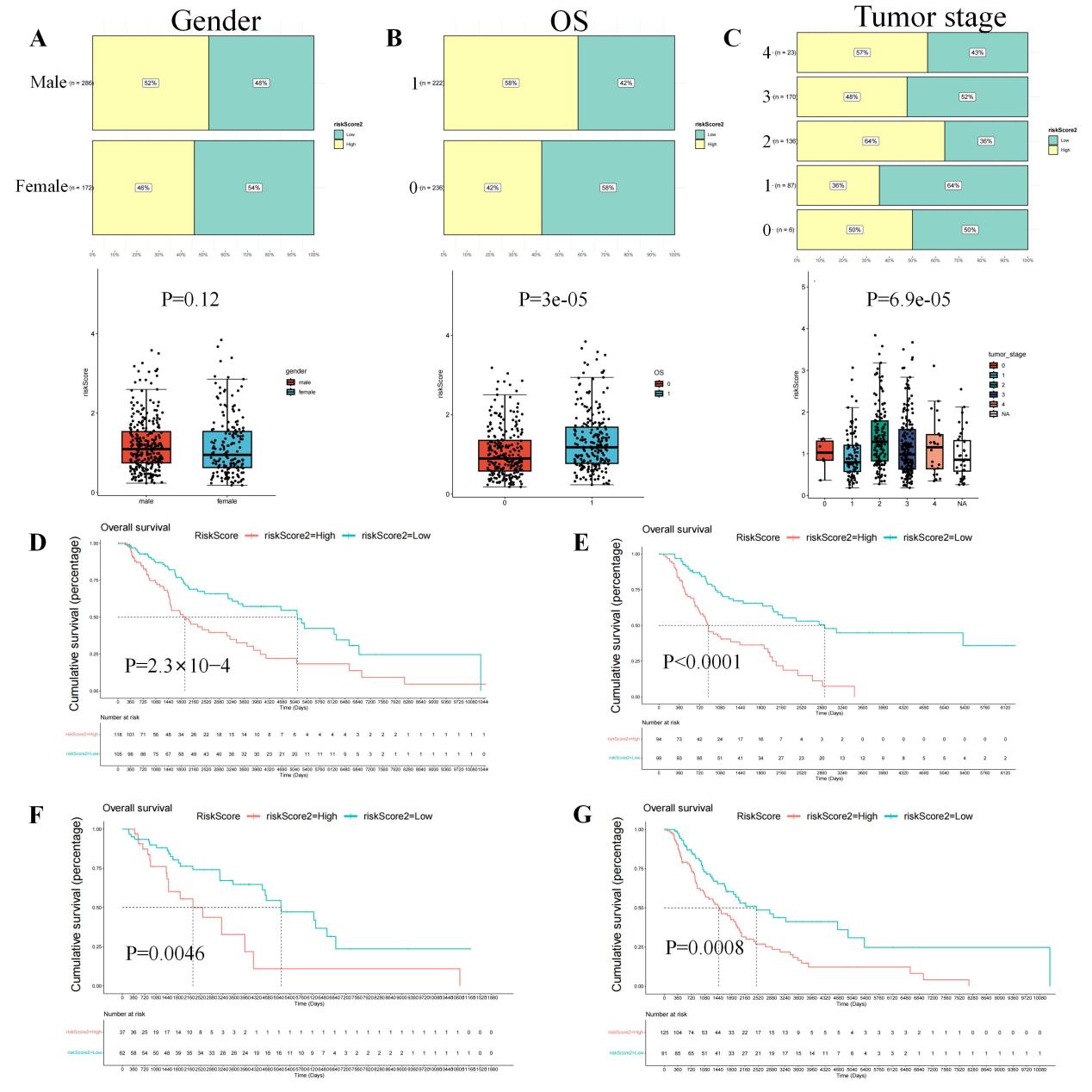


**Supplemental Fig. 7 Validation of prognostic performance of Tregs signature with different clinical status.** Frequency of gender (**A**), overall survival (**B**), and tumor stages (**C**) between high- and low-risk subgroups. Kaplan–Meier curves of overall survival in early stage (**D**), advanced stage (**E**), Clark 1, 2, and 3 level (**F**), and Clark 4 and 5 level (**G**) patients based on risk score in TCGA cohort.


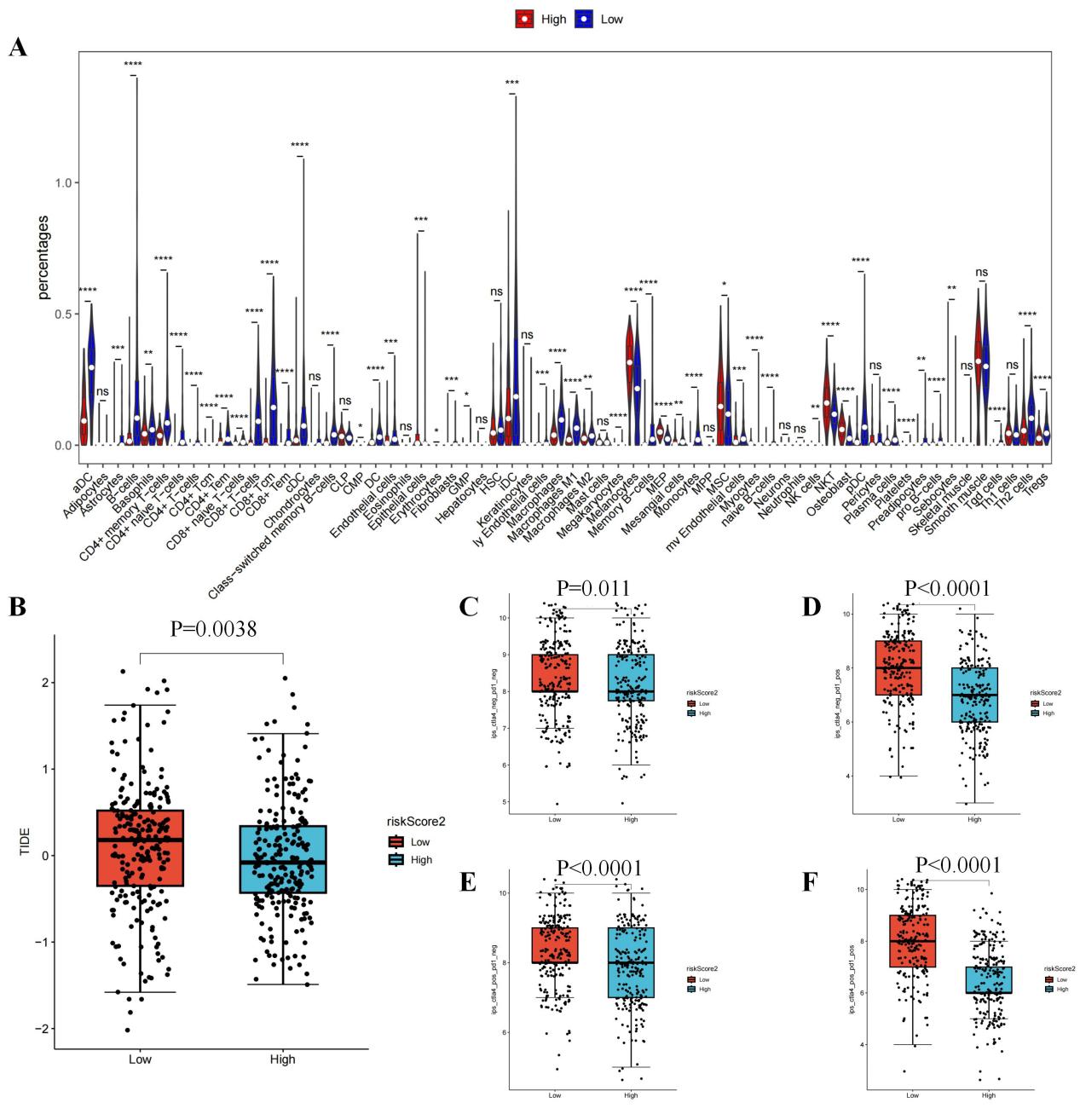


**Supplemental Fig. 8 Correlation of the Tregs signature with immune cell infiltration, TIDE score, and IPS score. (A)** The comparison of 64 infiltrating immune cells level between high-risk and low-risk groups. The TIDE scores **(B)** and IPS scores among ctla4_neg_pd1_neg group **(C)**, ctla4_neg_pd1_pos group **(D)**, ctla4_pos_pd1_neg group **(E)**, and ctla4_pos_pd1_pos group **(F)** were compared between high-risk and low-risk groups.


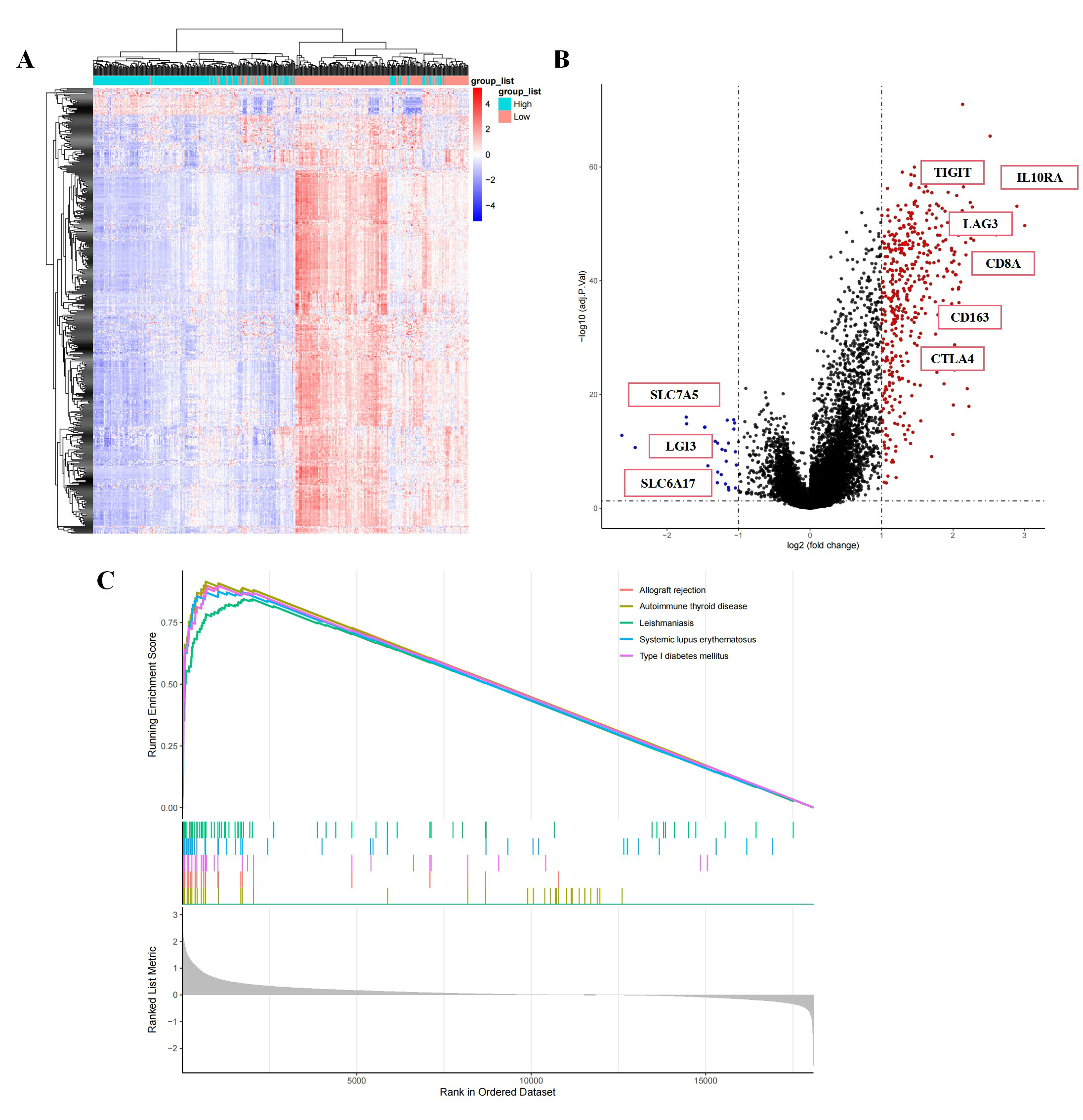


**Supplemental Fig. 9 Functional enrichment analysis of the genes that are closely correlated with the Tregs signature (Treg.Sig).** (**A**) Heatmap showed the 466 genes that had the most significant correlations with the signature. (**B**) Volcano plot mapped 438 positively correlated and 28 negatively correlated genes related to the signature. (**C**) GSEA analysis of the identified genes.


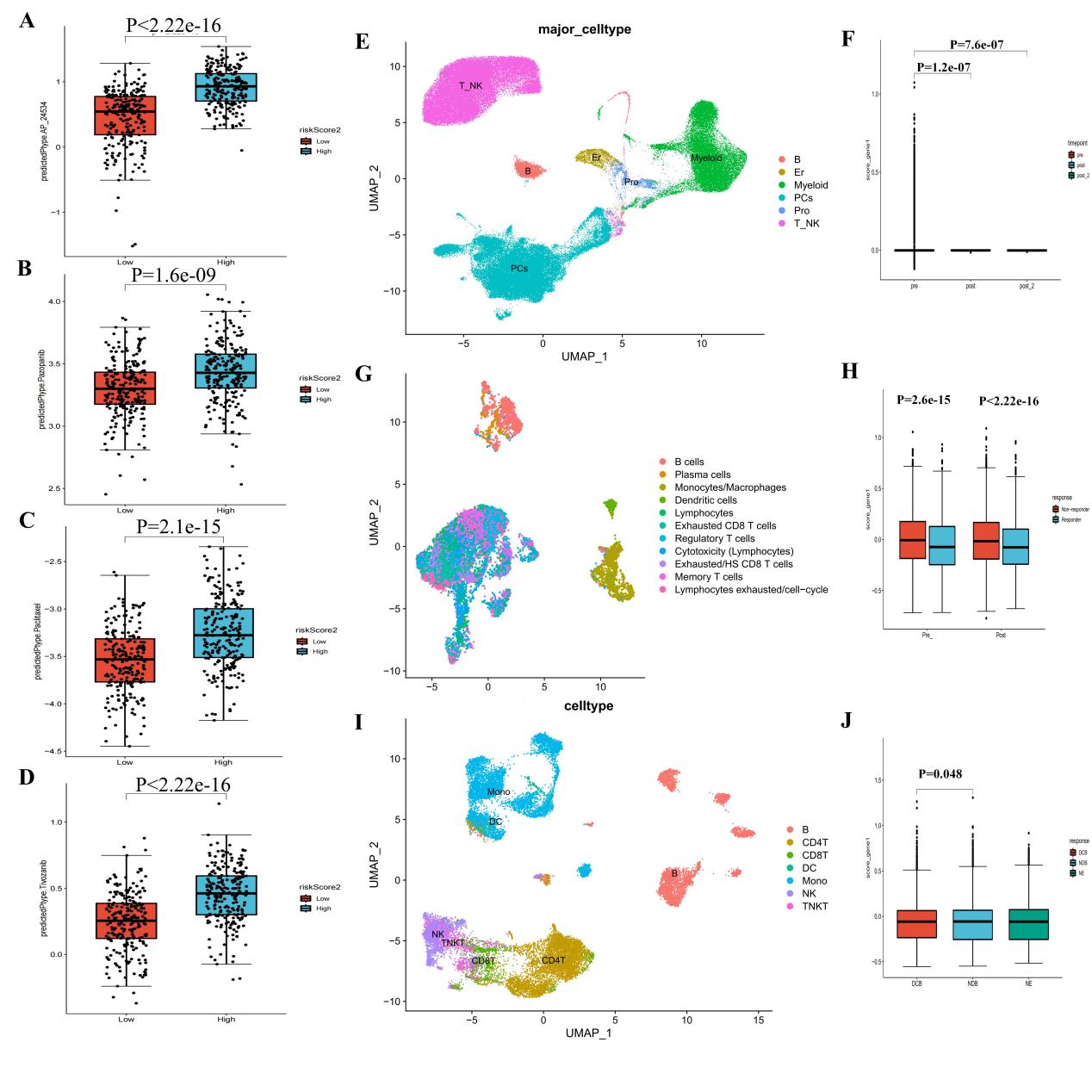


**Supplemental Fig. 10 The comparison of chemotherapy and immune checkpoint inhibitors response between high- and low-risk Treg.Sig groups.** Chemotherapy drugs AP_24534 (**A**), Pazopanib (**B**), Paclitaxel (**C**), and Tivozanib (**D**) therapy response between high- and low-risk subgroups. Single-cell RNA-sequencing analysis for predicting the probability of clinical resistance to immune checkpoint inhibitors in GSE161801 (**E, F**), GSE120575 (**G, H**), and GSE189125 (**I, J**) based on Treg.Sig.


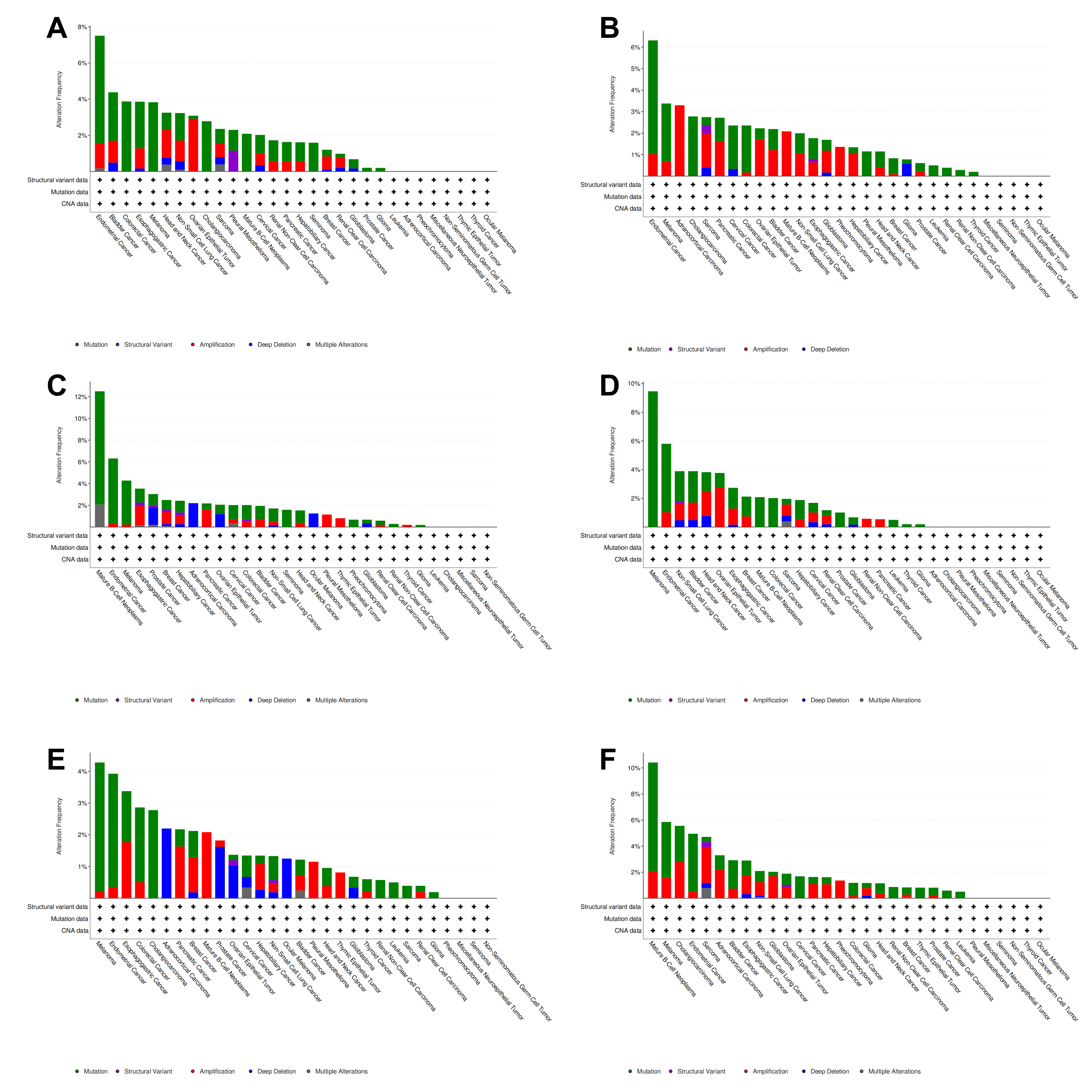


**Supplemental Fig. 11 The mutation frequencies of STAT subtype in melanoma.** (**A**) STAT1 mutation frequency. (**B**) STAT2 mutation frequency. (**C**) STAT3 mutation frequency. (**D**) STAT4 mutation frequency. (**E**) STAT5A mutation frequency. (**F**) STAT6 mutation frequency.
